# Supplementary material for: Tad pili with adaptable tips mediate contact-dependent killing during bacterial predation
Source: Nat Commun. 2025 May 13;16:4425. doi: 10.1038/s41467-025-58967-0 (PMC12075869; doi:10.1038/s41467-025-58967-0)
Supplement: Supplementary file 1 — Supplementary Information [file 41467_2025_58967_MOESM1_ESM.pdf]

Supplementary Figures and Legends

Tad-pili with adaptable tips mediate contact-dependent killing during bacterial predation

Julien Herrou, Laetitia My, Caroline Monteil, Marine Bergot, Rikesk Jain, Emmanuelle Martinez, Tâm Mignot.

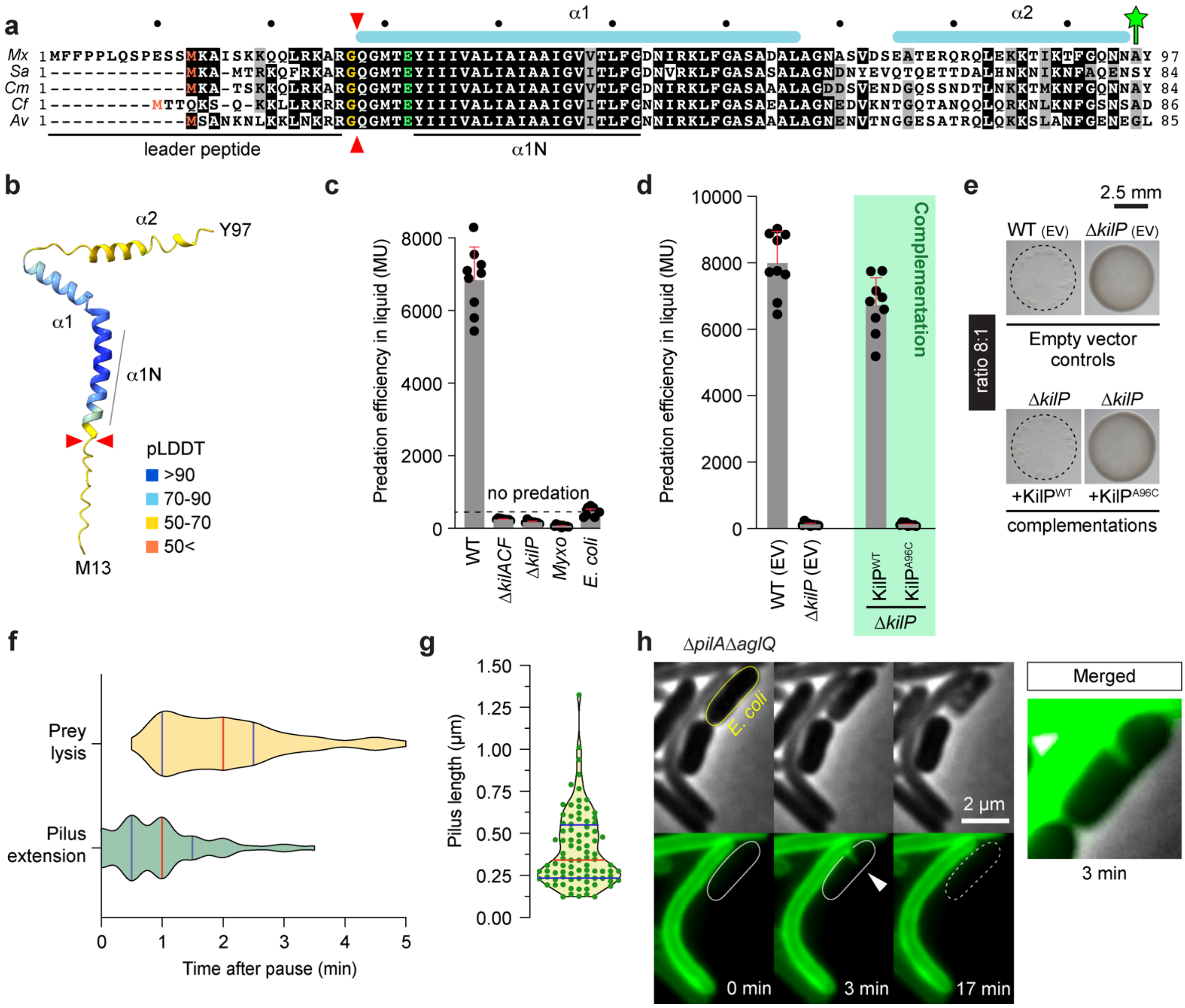

Supplementary Figure 1. KilP is the Kil major pilin.

a Amino acid sequence alignment of *Myxococcus* KilP (Mx, Mxan_5121) with orthologs identified in *Stigmatella aurantiaca* (Sa, STAUR_5888), *Corallococcus macrosporus HW-1* (Cm, LILAB_33575), *Cystobacter fuscus* (Cf, CYFUS_006936) and *Archangium violaceum* (Av, JQX13_32595). In these sequences, the predicted initiator methionine is highlighted in red. KilP secondary structure is shown above the alignment. The predicted cleavage site of the leader peptide is indicated by two red triangles. The hydrophobic α1N region is also delimited. To label KilP pilus with Alexa Fluor 488 C_5 maleimide (AF488mal), alanine 96 (indicated by a green star) was substituted by a cysteine. **b** The AlphaFold structure prediction of KilP is color-coded based on the predicted Local Distance Difference Test (pLDDT) corresponding to a per-residue model confidence score ranging from 0 (orange, low confidence) to 100 (dark blue, high confidence). **c** The predation ability of the Δ*kilP* strain is impaired in liquid CF after 24 hours of incubation. This bar graph represents the kinetics of CPRG hydrolysis catalyzed by the β-Galactosidase released in the supernatant by *E. coli* when lysed by *Myxococcus* wild-type (WT), Δ*kilACF* or Δ*kilP* strains. *Myxococcus* and *E. coli* alone were used as controls. **d** Ectopic expression of a wild-type *kilP* allele (M13-Y97) from a pSWU19-*PpilA* plasmid successfully complements a Δ*kilP* strain for predation on *E. coli* in liquid CF. However, the *kilP*^{A96C} mutant allele fails to complement this strain. The WT and Δ*kilP* strains transformed with the pSWU19 empty vector (EV) were used as controls in this assay. In panels **c** and **d**, experiments were performed in triplicate and repeated independently three times (*n*= 9 per strain). Error bars represent the standard deviation of the mean. MU = Miller Units. **e** Similarly, ectopic expression of the *kilP*^{WT} allele successfully complemented a motile Δ*kilP* strain for predation over *E. coli* on CF 1.5% agar plates supplemented with 0.075% glucose. However, the *kilP*^{A96C} mutant allele failed to complement this strain. Again, the empty vector (EV) strains were used as controls. In this assay, the prey and predator cells were mixed to an 8:1 ratio and pictures of the *E. coli* colonies were captured after 24 hours of predation. **f** By microscopy, we quantified the time interval between prey-induce motility arrest, KilP polymerization and prey lysis. The number of events us for quantification was *n*= 58. **g** The KilP pilus length distribution upon prey contact was also determined by microscopy. The number of events us for quantification was *n*= 88. In panels **f** and **g**, the red line represents the median, the blue lines represent the upper and lower quartiles. **h** In a non-motile strain (Δ*pilA*Δ*aglQ*), a labeled KilP^{A96C} pilus can still be polymerized at the prey contact site and it correlates with prey lysis. Source data are provided as a Source Data file.

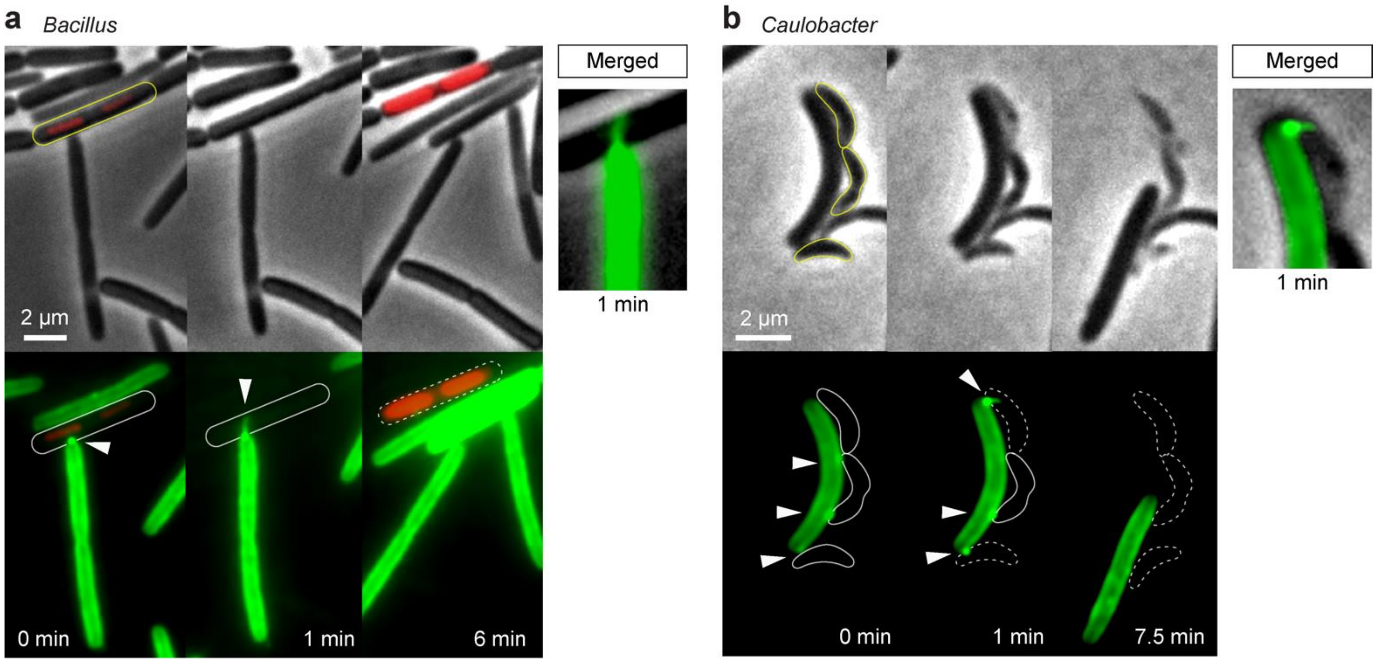

Supplementary Figure 2. The KilP pilus polymerizes upon contact with different bacterial species.
a Microscopy images showing a KilP^{A96C} pilus (white arrowhead) labeled with AF488mal extending at the contact site with *B. subtilis*. Since *B. subtilis* lysis is slow, we supplemented the agar pad with 30 μM propidium iodide to facilitate the visualization of dead cells (red fluorescence). **b** Similarly, polymerization of the KilP pilus (white arrowheads) can be observed at the contact sites with *C. crescentus* and it correlates with prey lysis.

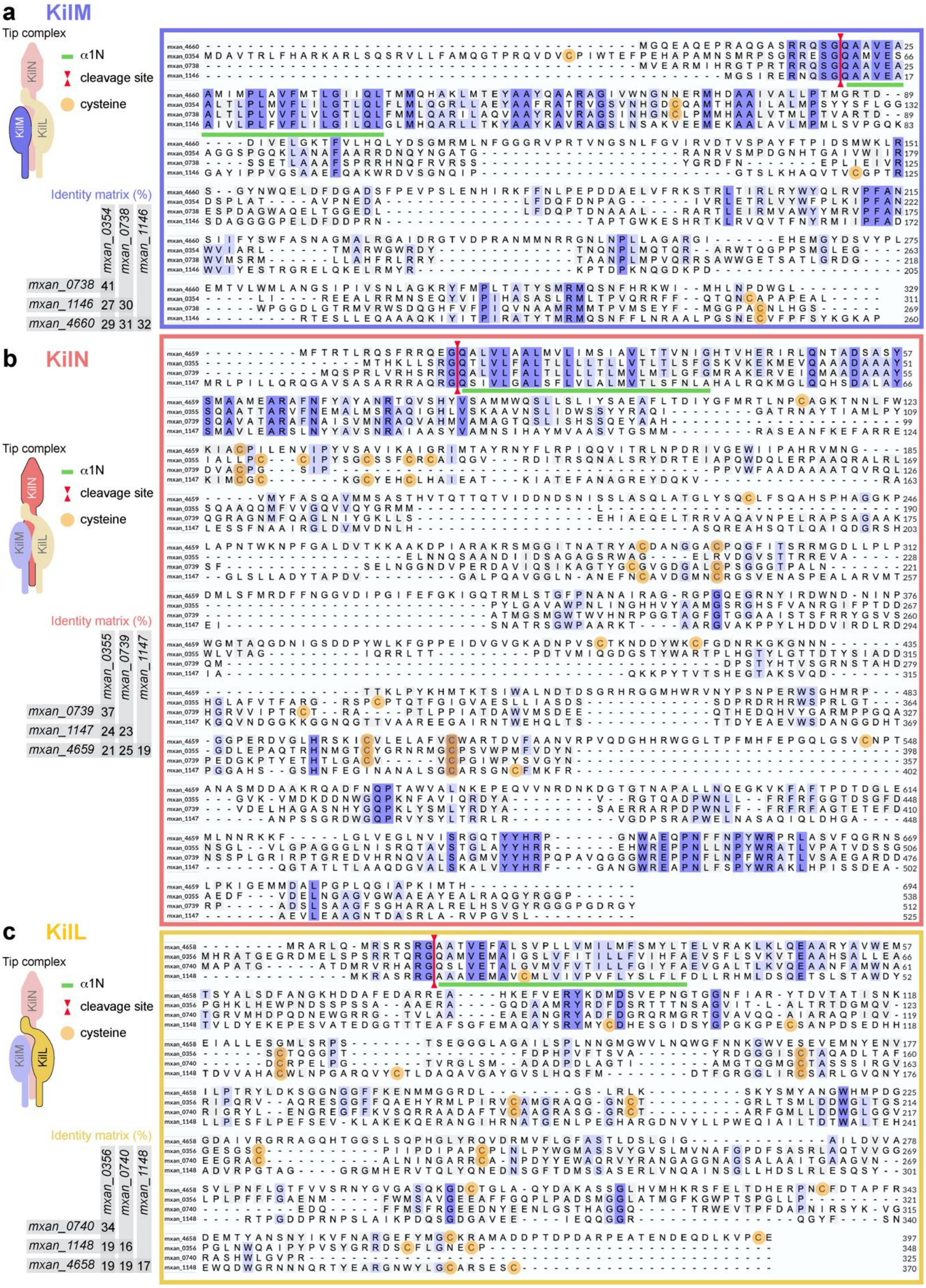

Supplementary Figure 3. Multiple amino acid sequence alignments between the different minor pilins constituting the Tips.
a Multiple amino acid sequence alignment between the different KilM paralogs (Mxan_0354, Mxan_0738, Mxan_1146 and Mxan_4660). **b** Multiple amino acid sequence alignment between the different KilN paralogs (Mxan_0355, Mxan_0739, Mxan_1147 and Mxan_4659). **C** Multiple amino acid sequence alignment between the different KilL paralogs (Mxan_0356, Mxan_0740, Mxan_1148 and Mxan_4658). The percentage of identity between each paralog is provided in the corresponding identity matrix. Cysteines are highlighted by orange circles in the alignments.

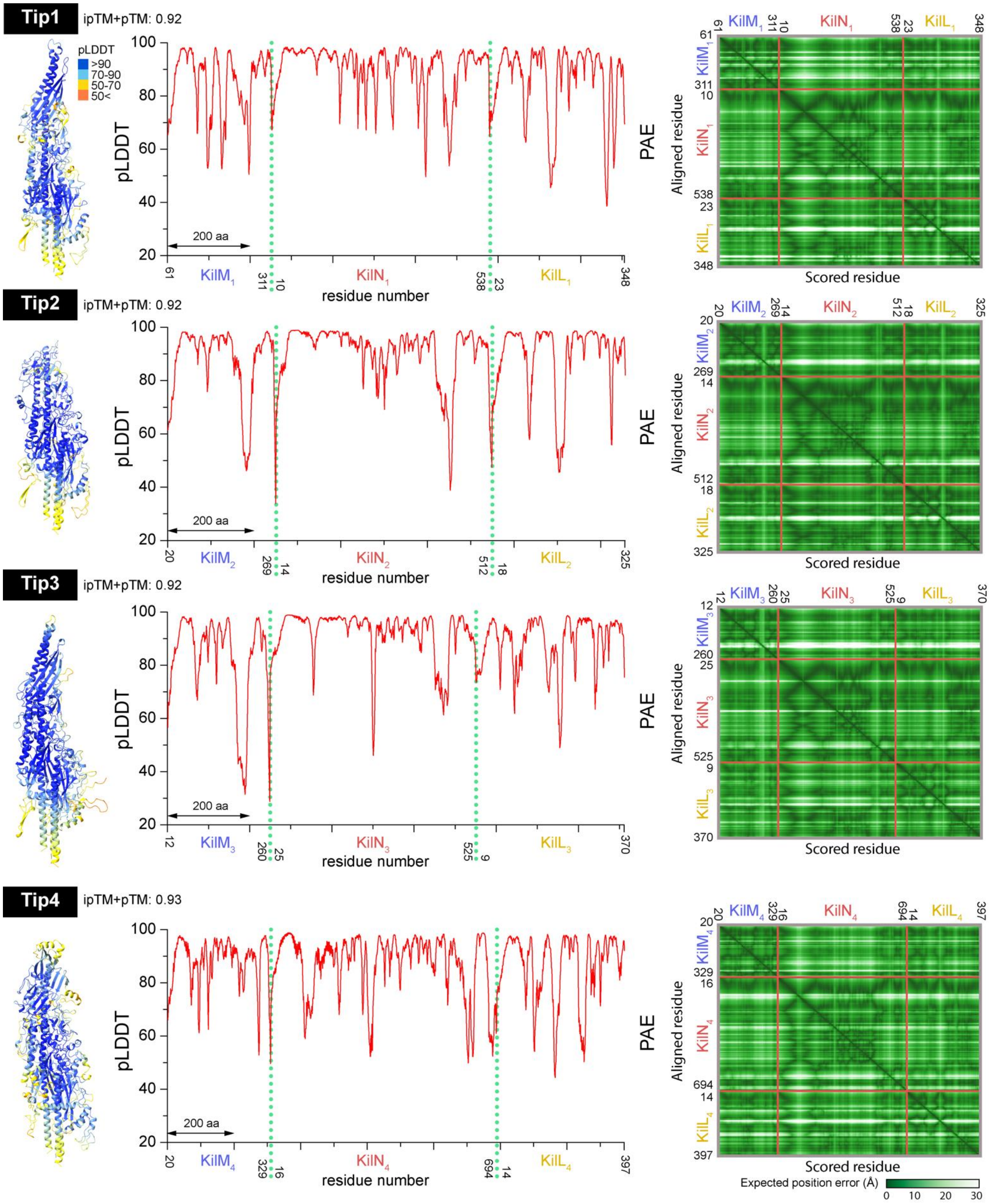

Supplementary Figure 4. Predicted Template Modeling scores (pTM), interface predicted Template Modeling scores (ipTM), predicted Local Distance Difference Tests (pLDDT) and Predicted Aligned Errors (PAE) of the Tips AlphaFold predictions.

To generate these AlphaFold models, the leader peptide regions were systematically removed from the minor pilin amino acid sequences. The predicted structures are color-coded based on the pLDDT corresponding to a per-residue model confidence score: disordered (50<); low confidence (50-70); good confidence (70-90); high confidence (>90). PAE scores: low PAE for residue pairs x, y from two different proteins or domains indicates that AlphaFold predicted well-defined relative positions and orientations for these proteins or domains. On the contrary, if PAE is high, then the relative positions and/or orientations of these proteins or domains in the 3D structure are uncertain and should not be interpreted. ipTM+pTM score: the closer to one, the greater the overall confidence for the multimer model.

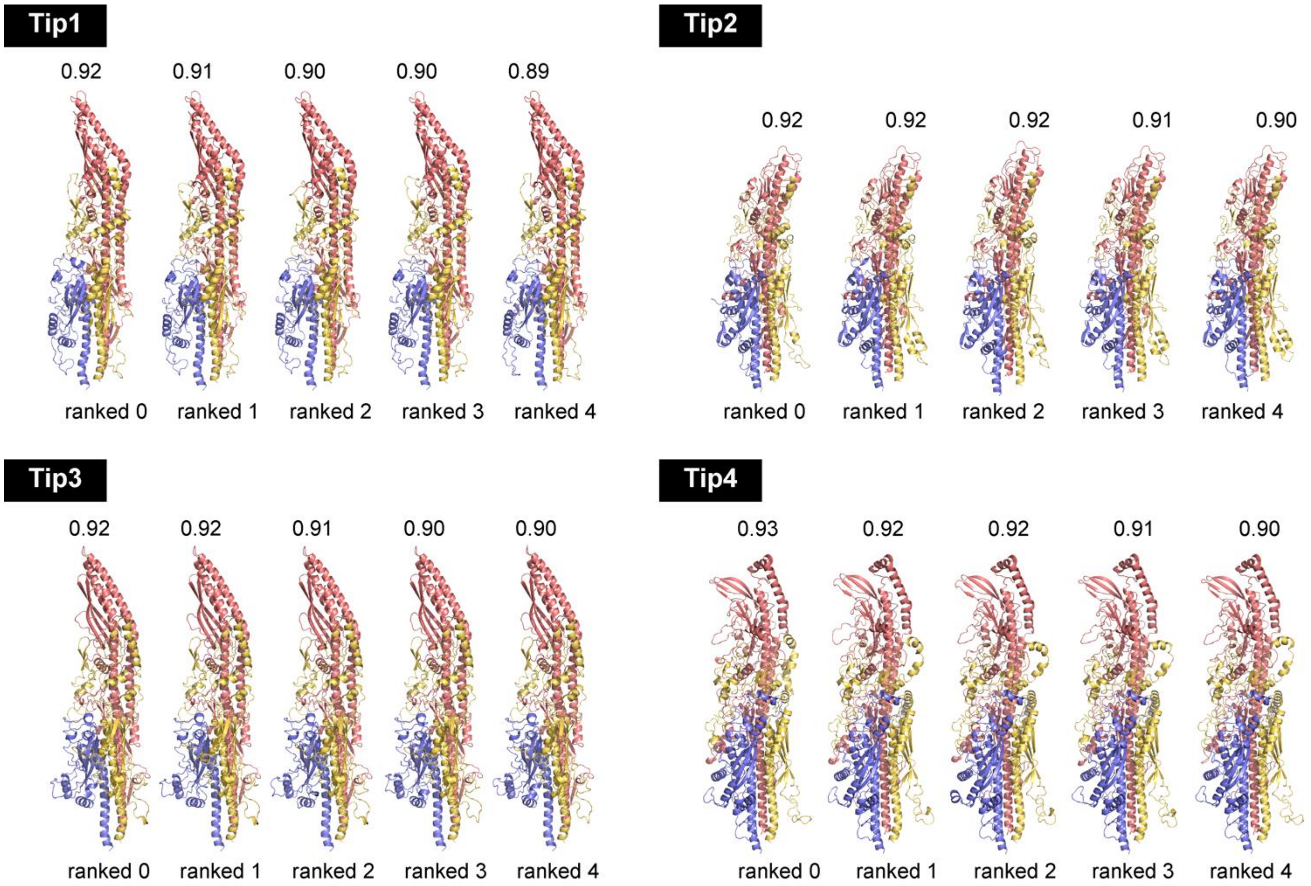

Supplementary Figure 5. Ranked AlphaFold predictions of the different KilLMN Tip complexes.
To generate these AlphaFold models, the leader peptide regions were systematically removed from the minor pilin amino acid sequences. For a specific Tip, all five generated models are nearly identical, suggesting that these predictions are reliable. The corresponding ipTM+pTM scores are indicated above each complex.

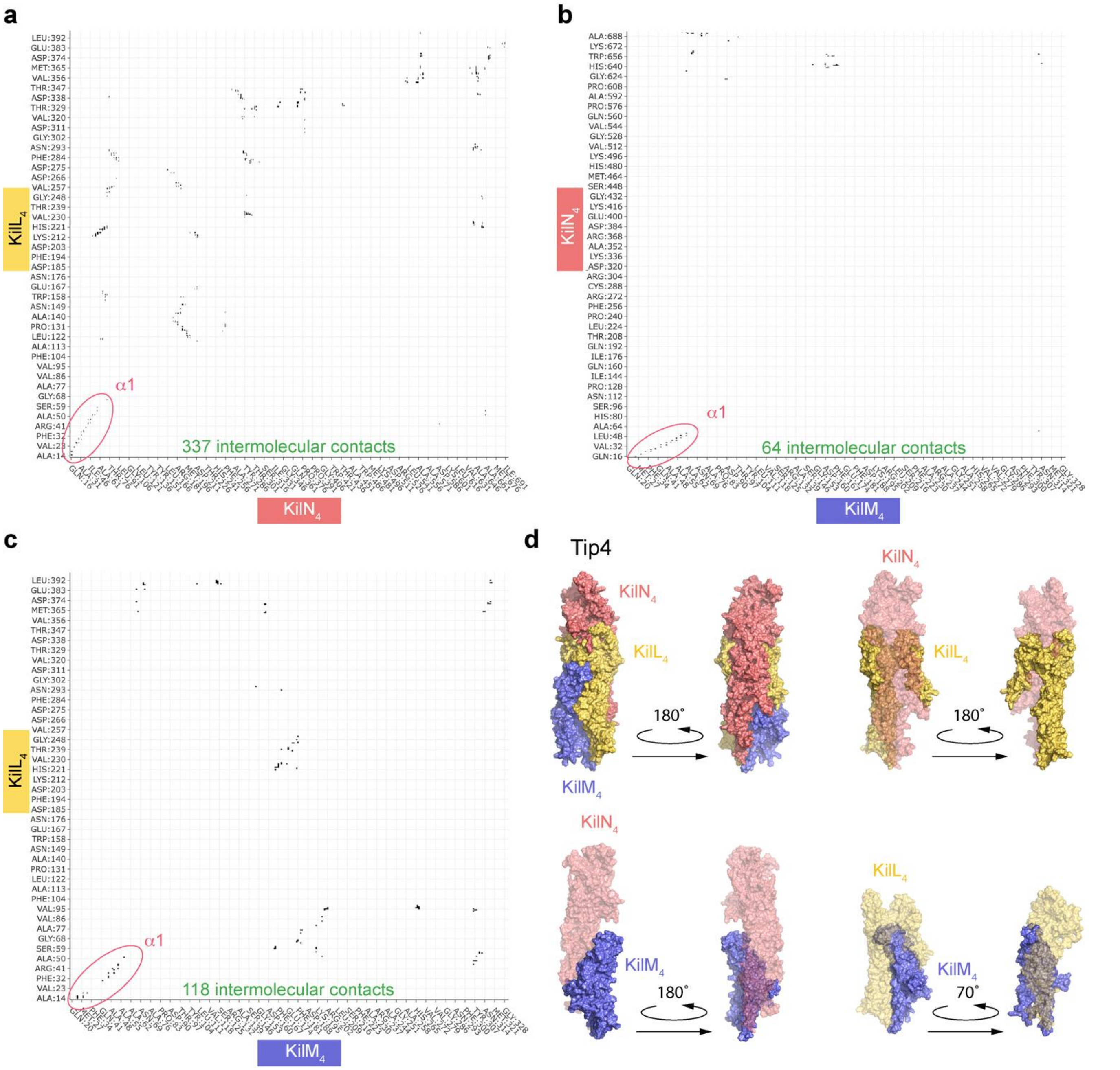

Supplementary Figure 6. KilL$_4$, KilM$_4$ and KilN$_4$ interact to form the heterotrimeric Tip4 complex.
a Contact map between KilL$_4$ and KilN$_4$ (cutoff 4 Å, 337 intermolecular contacts). **b** Contact map between KilN$_4$ and KilM$_4$ (cutoff 4 Å, 64 intermolecular contacts). **c** Contact map between KilL$_4$ and KilM$_4$ (cutoff 4 Å, 118 intermolecular contacts). In each map, the intermolecular contacts corresponding to the α1N-bundle are highlighted by a red ellipse. **d** Distinct surfaces of interaction enable KilL$_4$, KilM$_4$ and KilN$_4$ to assemble and form the Tip4 complex.

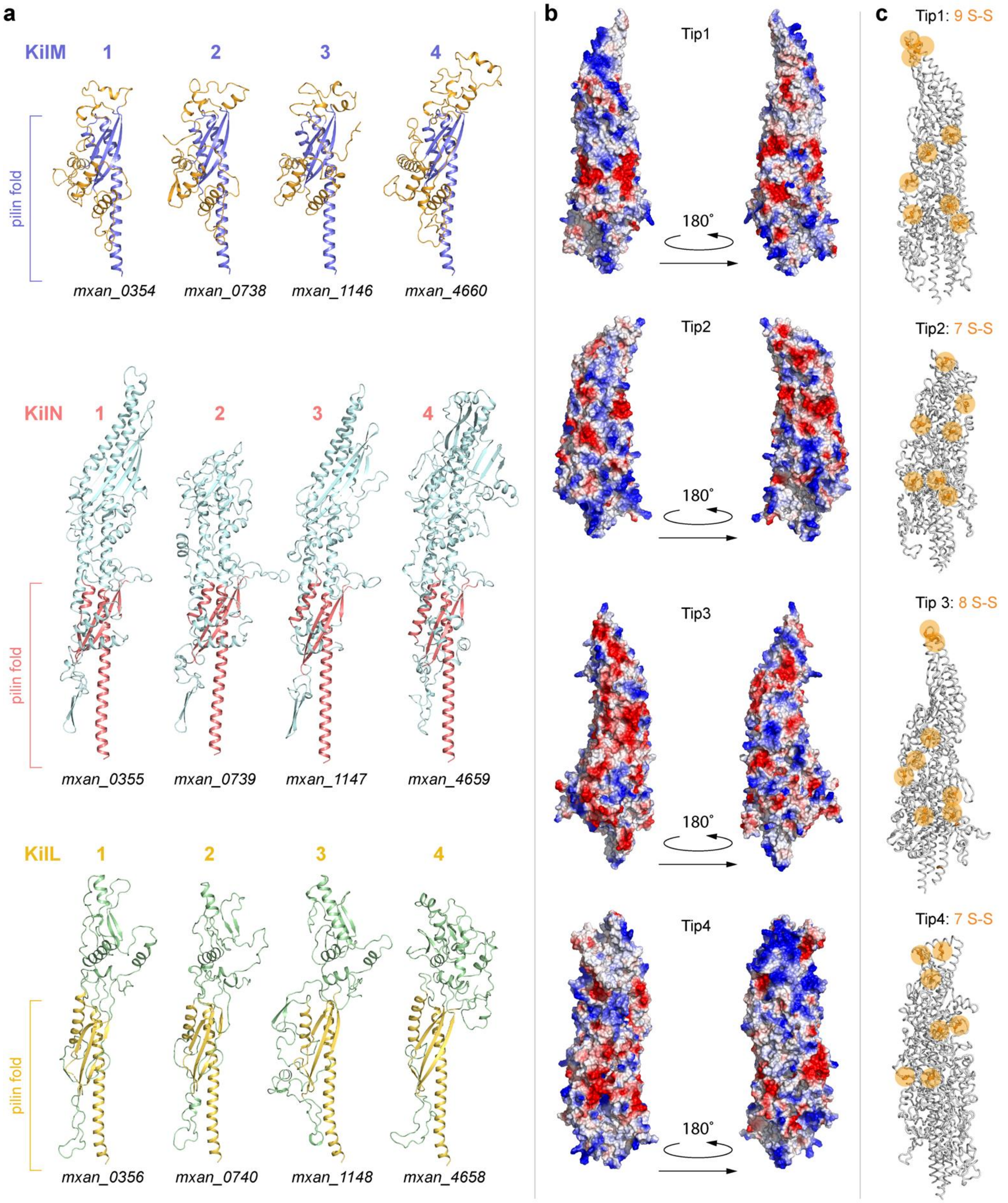

Supplementary Figure 7. The four Tips correspond to distinct minor pilin heterotrimeric complexes.
a AlphaFold predictions of the KilM, KilN and KilL minor pilin paralogs constituting the four different Tips. The conserved pilin folds and the modular domains are delimited in different colors. To generate these AlphaFold predictions, the leader peptide regions were systematically removed from the minor pilin amino acid sequences. **b** Representation of the electrostatic surface potentials of the different Tips (positively charged regions are in blue and negatively charged regions are in red). **c** The different Tips present many cysteines potentially involved in the formation of disulfide bonds (highlighted by orange circles).

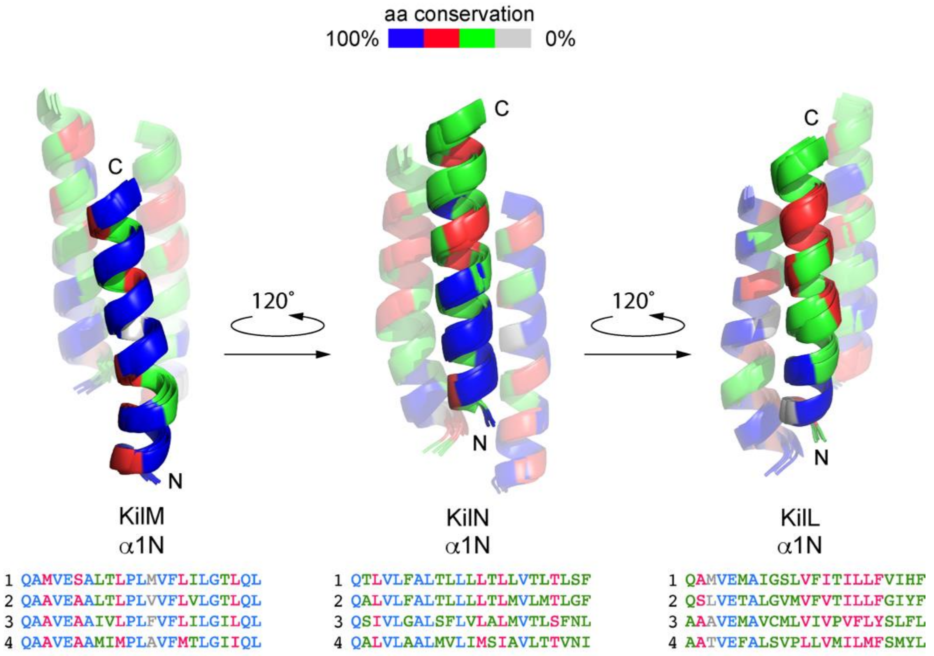

Supplementary Figure 8. Amino acid conservation of the Tip1, Tip2, Tip3 and Tip4 α1N-bundles.
The corresponding regions were structurally aligned and residues were color-coded based on their conservation scores.

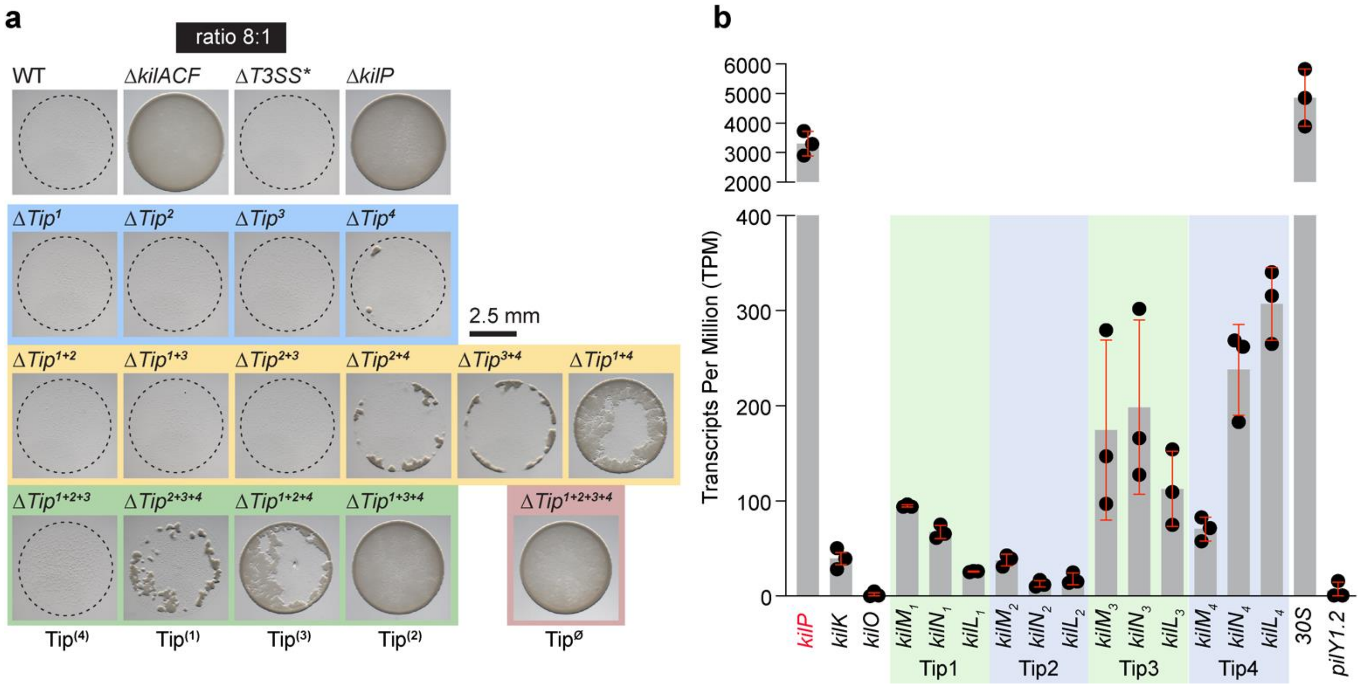

Supplementary Figure 9. The different Tip complexes play an essential role in predation.
a This panel shows representative pictures used to measure the surface of *E. coli* colony killed by the different Tip mutants after 24 hours of predation. In this assay, prey and predator cells were mixed to an 8:1 ratio and spotted on CF 1.5% agar plates supplemented with 0.075% glucose. All the strains used in this assay were motile. **b** A RNA-seq analysis was used to determine the expression levels of the *tip*-gene clusters (*kilLMN*), *kilP, kilK* and *kilO* during predation on CF agar plates, using *E. coli* as prey. The Ribosomal 30S and minor pilin PilY1.2 encoding genes were used as references for high and low expression levels, respectively. Data were extracted and computed from Jain R. *et al.* (2025) PNAS,122 (5) e2420875122. For each gene, data is compiled from three independent biological replicates (*n*= 3 per gene). Error bars represent the standard deviation of the mean. Source data are provided as a Source Data file.

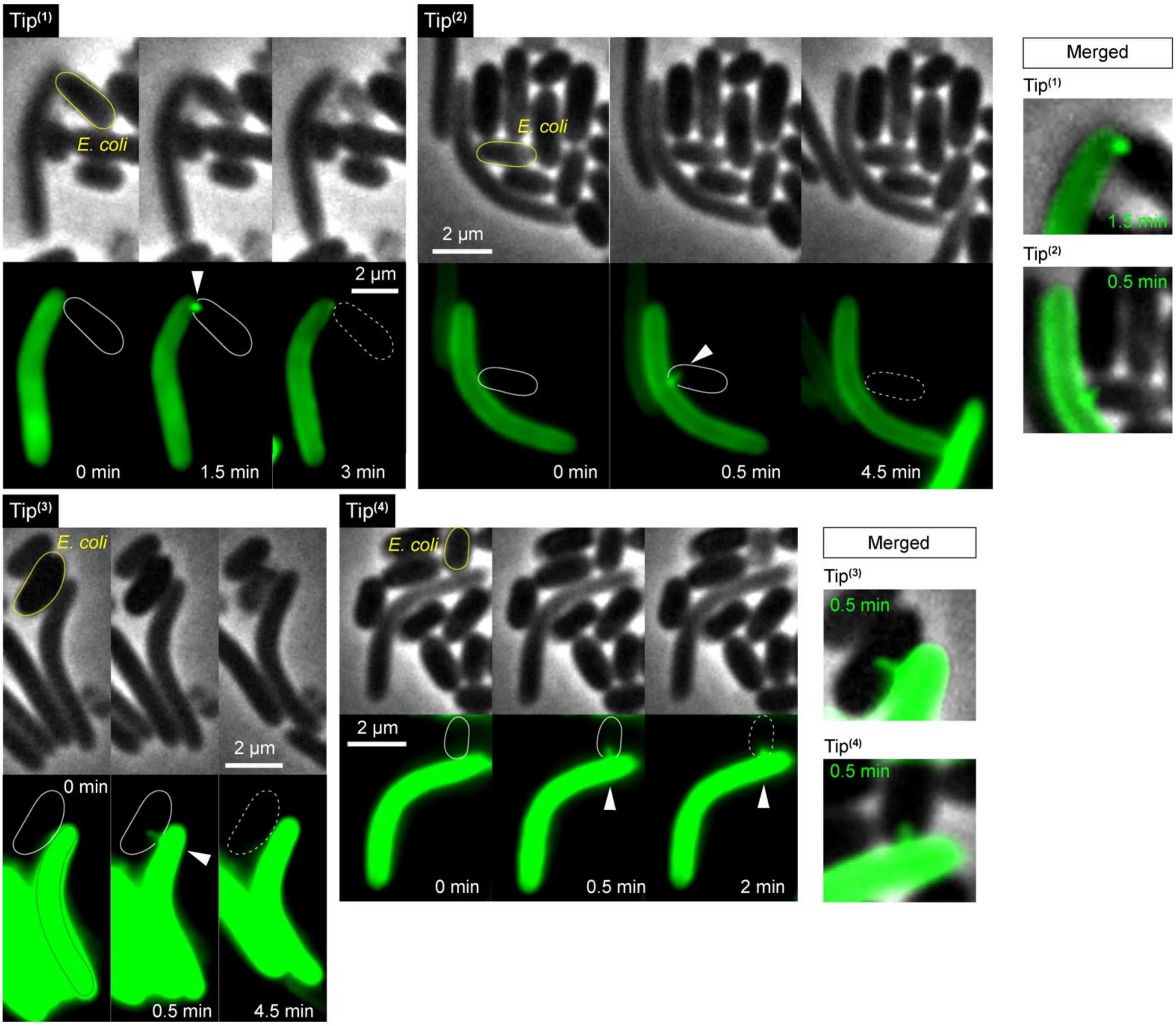

Supplementary Figure 10. Each Tip complex can initiate the polymerization of the KilP pilus.
Microscopy images demonstrating that the different single-Tip expressing strains can polymerize a KilP^{A96C} pilus labeled with AF488mal (white arrowheads). It correlates with motility pausing and prey cell lysis.

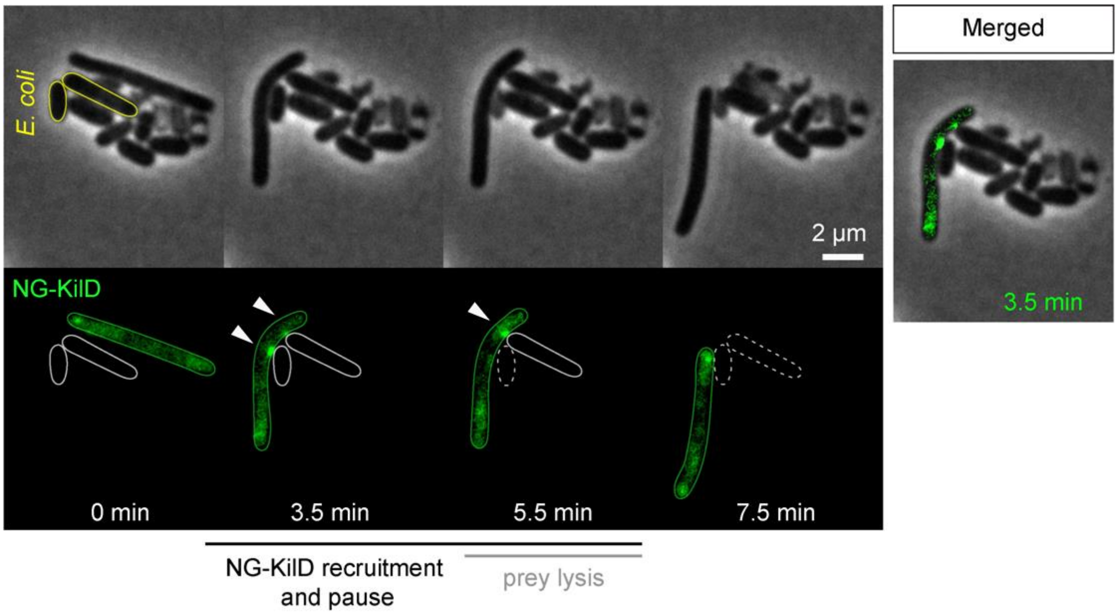

Supplementary Figure 11. The Kil system is assembled at the prey contact site.
Upon contact with prey, *Myxococcus* temporarily halts its motility and recruits NG-KilD at the prey contact site (green fluorescent clusters, white arrowheads). This recruitment is immediately followed by prey lysis. After prey cell death, motility resumes.

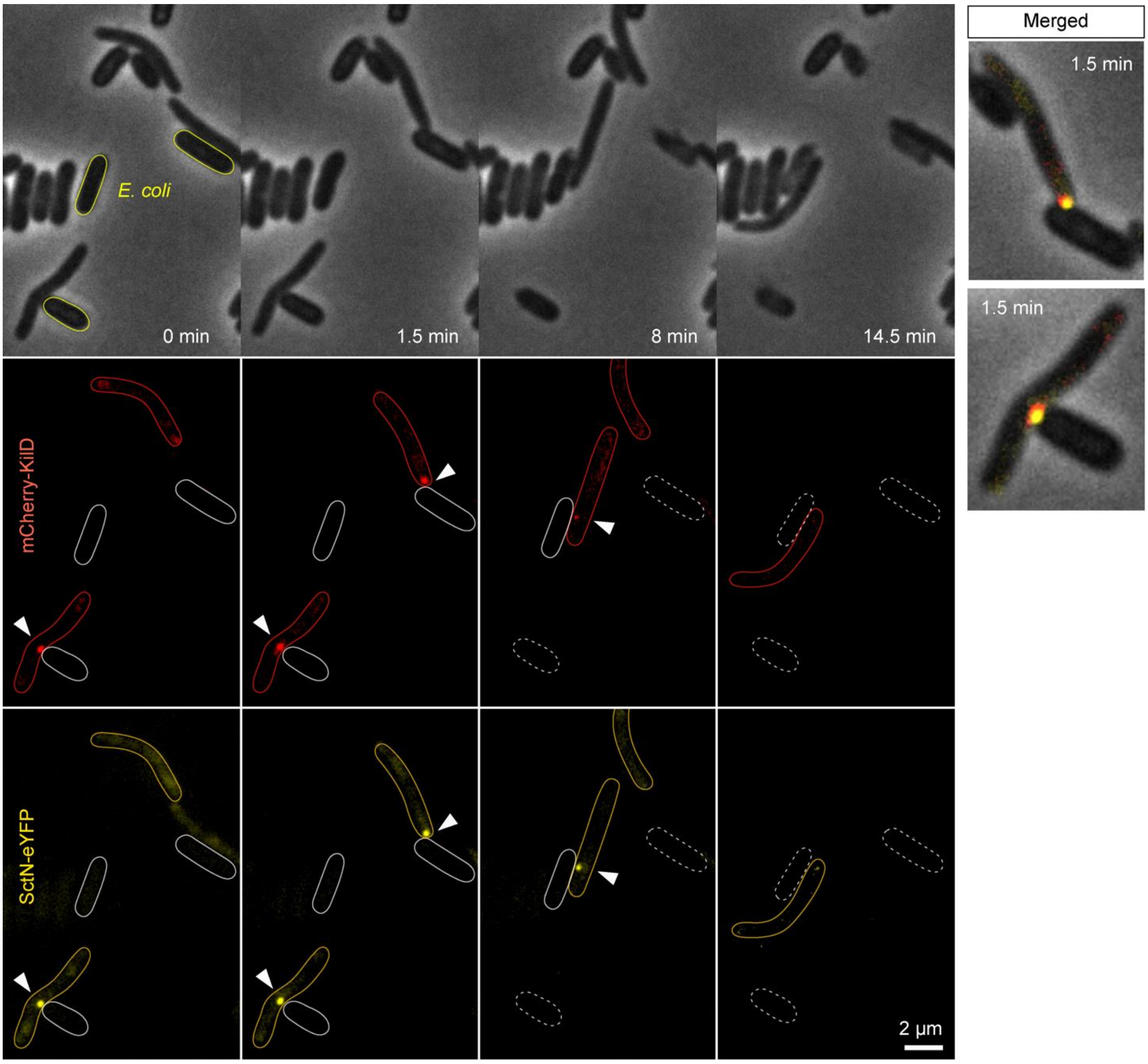

Supplementary Figure 12. The Kil system and the T3SS* colocalize at the prey contact site.
By fluorescence microscopy, we observed that mCherry-KilD clusters (corresponding to the Kil system assembly in red, white arrowheads) and SctN-eYFP clusters (corresponding to the "needleless" T3SS* ATPase in yellow, white arrowheads) are simultaneously recruited at the prey contact site, and it correlates with motility pausing, prey killing and lysis.

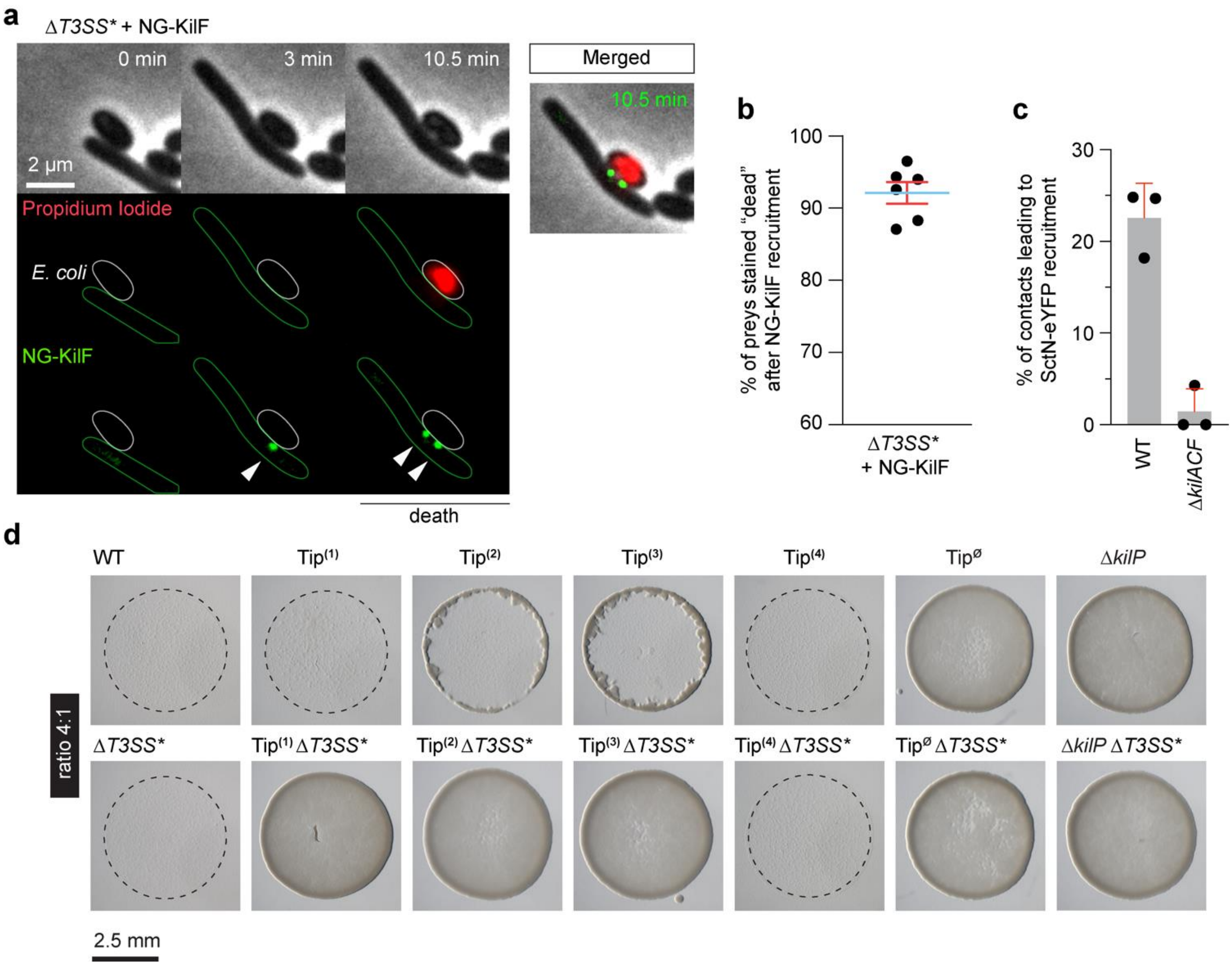

Supplementary Figure 13. Tip1, Tip2 and Tip3 predation activities require a functional T3SS*

a Microscopy images showing that the Kil system is still assembled at the prey contact site in a Δ*T3SS** strain. Upon contact with prey, *a* Δ*T3SS** strain halts its motility and recruits NG-KilF (green fluorescent clusters, white arrowheads). Prey killing is confirmed by staining dead prey cells with propidium iodide (red fluorescence). **b** In a Δ*T3SS** strain, we quantified the percentage of *E. coli* cells stained "dead" after the recruitment of NG-KilF. The number of stained "dead" prey cell events used for quantification was *n*= 436. **c** T3SS* recruitment is impaired in a *kil* mutant. The percentage of contacts with *E. coli* leading to SctN-eYFP recruitment was quantified in motile WT and Δ*kilACF* strains. The number of prey contacts used for quantification was *n*= 471 for WT and *n*= 819 for Δ*kilACF*. The number of SctN-eYFP recruitment events was *n*= 105 for WT and *n*= 11 for Δ*kilACF*. In panels **b** and **c**, quantifications were obtained from six and three independent 30-minute microscopy movies, respectively. Error bars represent the standard deviation of the mean. **d** The T3SS* is functionally associated to Tip1, Tip2 and Tip3. This panel shows representative pictures of *E. coli* / *Myxococcus* predation spots used to quantify the number of prey-CFUs that survived to predation after 5 hours on CF 1.5% agar plates supplemented with 0.075% glucose. In this assay, the different single-Tip expressing strains, carrying or not a *T3SS** deletion, were mixed with *E. coli* to a 4:1 prey-to-predator ratio. Pictures of the predation spots were taken after 24 hours of predation. Source data are provided as a Source Data file.

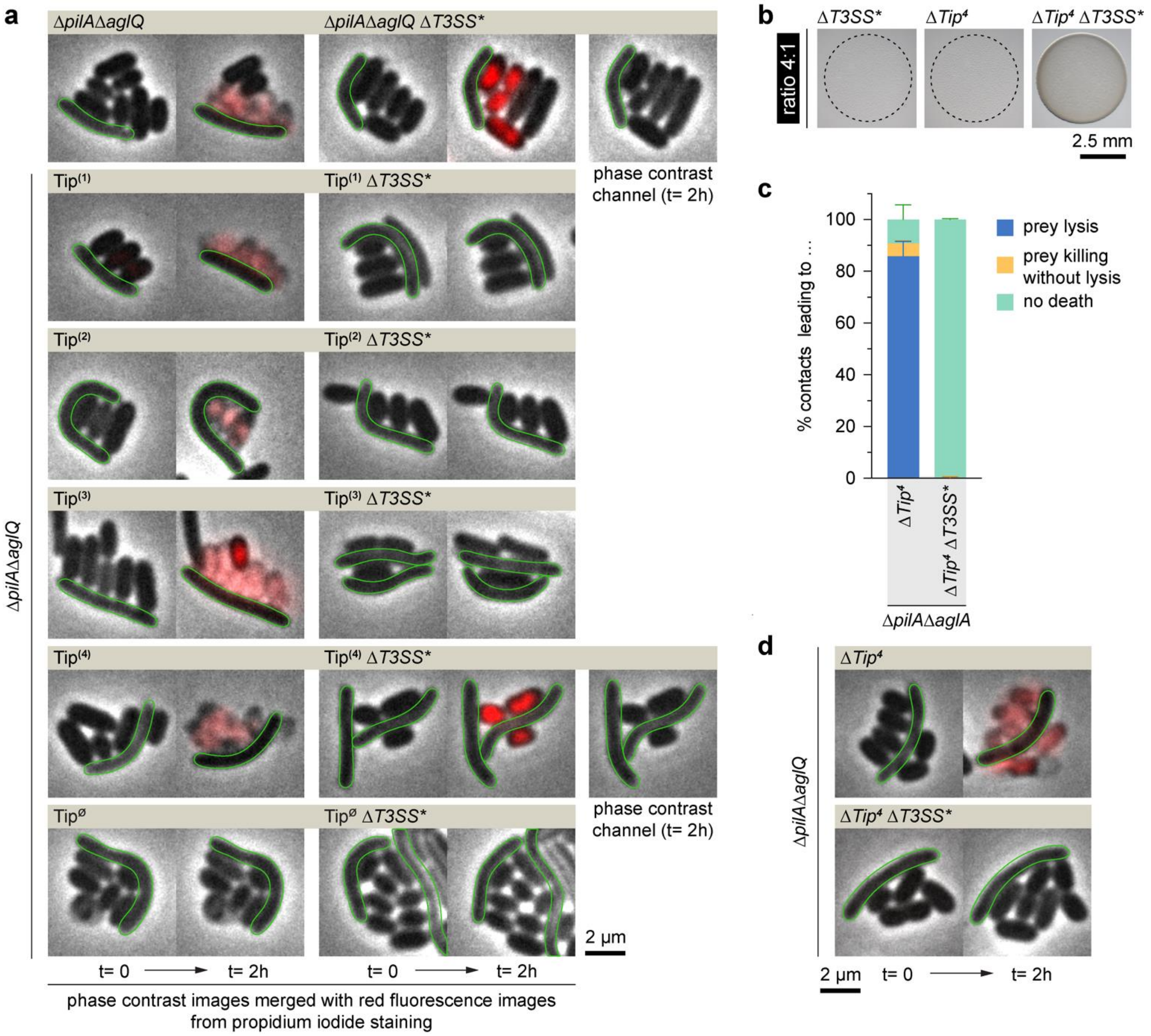

Supplementary Figure 14. Prey intoxication by Tip1, Tip2 or Tip3 requires the T3SS*.
a This panel shows representative images of *E. coli* cells in contact with non-motile strains expressing all Tips, one Tip only ($Tip^{(n)}$), or no Tips ($Tip^{\varnothing}$) and carrying or not a *T3SS** deletion. After 2-hour incubation on a microscopy pad supplemented with propidium iodide, "dead-but-not-lysed" *E. coli* cells were stained in red. With exception of Δ*T3SS** Tip4-expressing strain, no prey intoxication could be observed in the single-Tip expressing strains carrying a *T3SS** deletion. **b** Representative images showing that a motile $\Delta Tip^4 \Delta T3SS^*$ strain fails to predate *E. coli* on CF 1.5% agar plates supplemented with 0.075% glucose. This indicates that presence of Tip1, Tip2 and Tip3 is not sufficient for prey killing in absence of T3SS*. Images of *E. coli* colonies were acquired after 24 hours of incubation using a 4:1 prey-to-predator ratio. **c** After 2-hour exposure to non-motile ΔTip^4 strains, carrying or not a T3SS* deletion, we determined by microscopy the percentage of contacts leading to *E. coli* lysis (in blue), *E. coli* death without lysis (in yellow, death was confirmed with propidium iodide staining), and *E. coli* survival (in pale cyan). The number of prey contacts used for quantification was *n*= 505 for ΔTip^4 and *n*= 470 for $\Delta Tip^4 \Delta T3SS^*$. Quantifications were performed on three independent experiments. Error bars represent the standard deviation of the mean. **d** Representative images of *E. coli* cells in contact with non-motile ΔTip^4 ± Δ*T3SS** used for quantification in panel **c**. After 2-hour incubation on a microscopy pad supplemented with propidium iodide, "dead-but-not-lysed" *E. coli* cells were stained in red. In a $\Delta Tip^4 \Delta T3SS^*$ strain, no prey killing and lysis events were observed, confirming that the predation performances of Tip1, Tip2 and Tp3 is linked to the T3SS*. Source data are provided as a Source Data file.

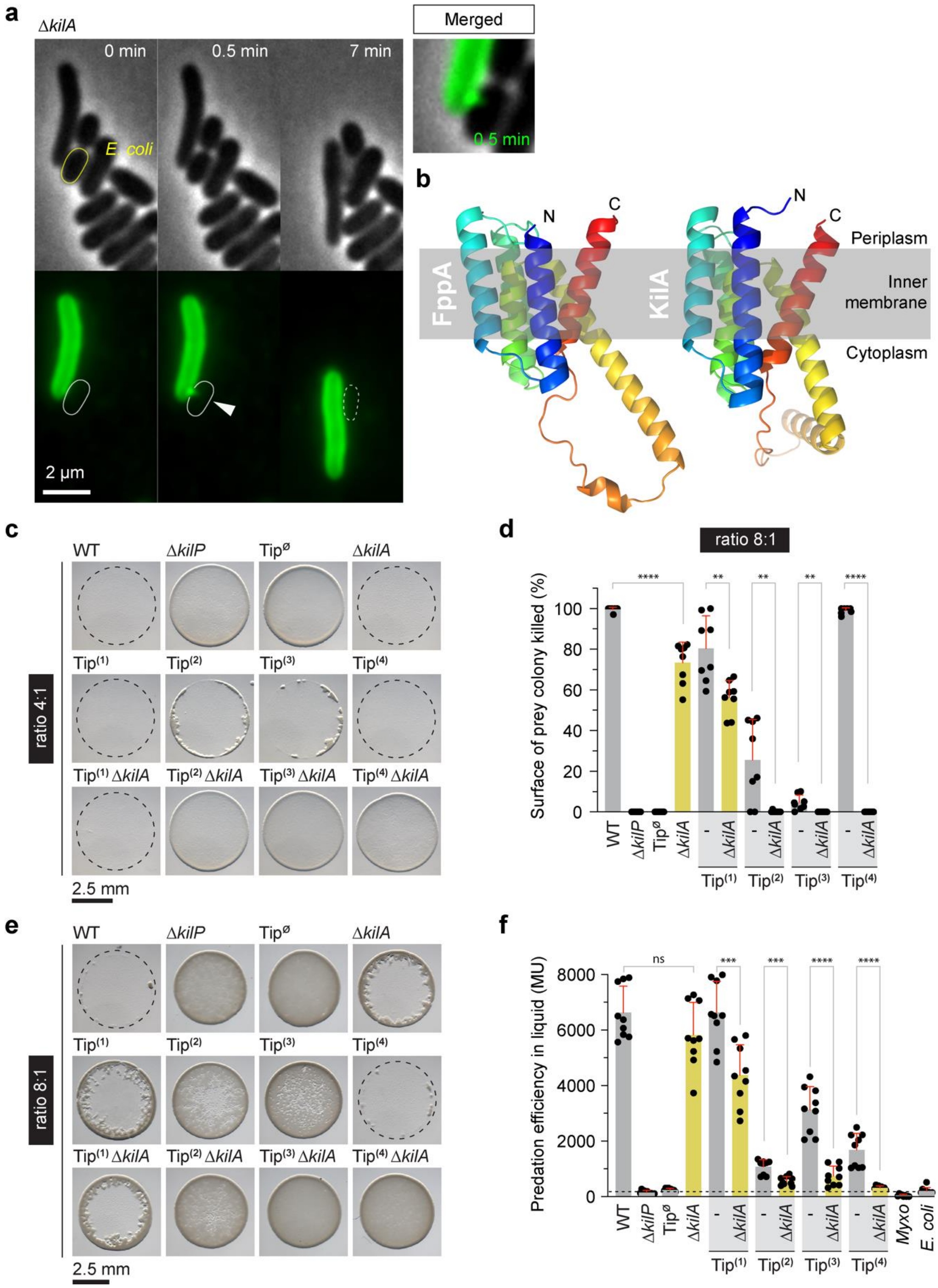

Supplementary Figure 15. The prepilin peptidase KilA is linked to the maturation of a subset of Tip complexes.
a Microscopy images demonstrating that polymerization of a fluorescently labeled KilP^{A96C} pilus (white arrowhead) is still occurring in a Δ*kilA* strain. **b** The AlphaFold predictions of *Pseudomonas* FppA (PA4295) and *Myxococcus* KilA (Mxan_3105) indicate that these prepilin peptidases present strong structural homologies. **c** This panel shows representative pictures used to measure the surface of *E. coli* colony killed by the different Δ*kilA* mutants after 24 hours of predation. In this assay, prey and predator cells were mixed to a 4:1 ratio and spotted on CF 1.5% agar plates supplemented with 0.075% glucose. All the *Myxococcus* strains used in this assay were motile. **d** The predation efficiency of the different Δ*kilA* mutants was also quantified on solid surfaces at an 8:1 prey-to-predator ratio. This experiment was performed independently four times, each data point representing a prey colony (*n*= 8 per strain). **e** This panel shows representative pictures used to quantify the predation efficiencies reported in panel **d**. **f** The predation efficiency of the different Δ*kilA* mutants was also evaluated in liquid CF after 24 hours of incubation. This graph represents the kinetics of CPRG hydrolysis catalyzed by the β-Galactosidase released in the supernatant from lysed *E. coli*. *Myxococcus* and *E. coli* alone were used as negative controls. This experiment was performed in triplicate and repeated independently three times (*n*= 9 per strain). In panels **d** and **f**, error bars represent the standard deviation of the mean. Statistics were determined using two-tailed unpaired t test. Significance: $^{ns}P>0.05$, $^{**}P<0.01$, $^{***}P<0.001$, $^{****}P<0.0001$. Source data and exact *p*-values are provided as a Source Data file.

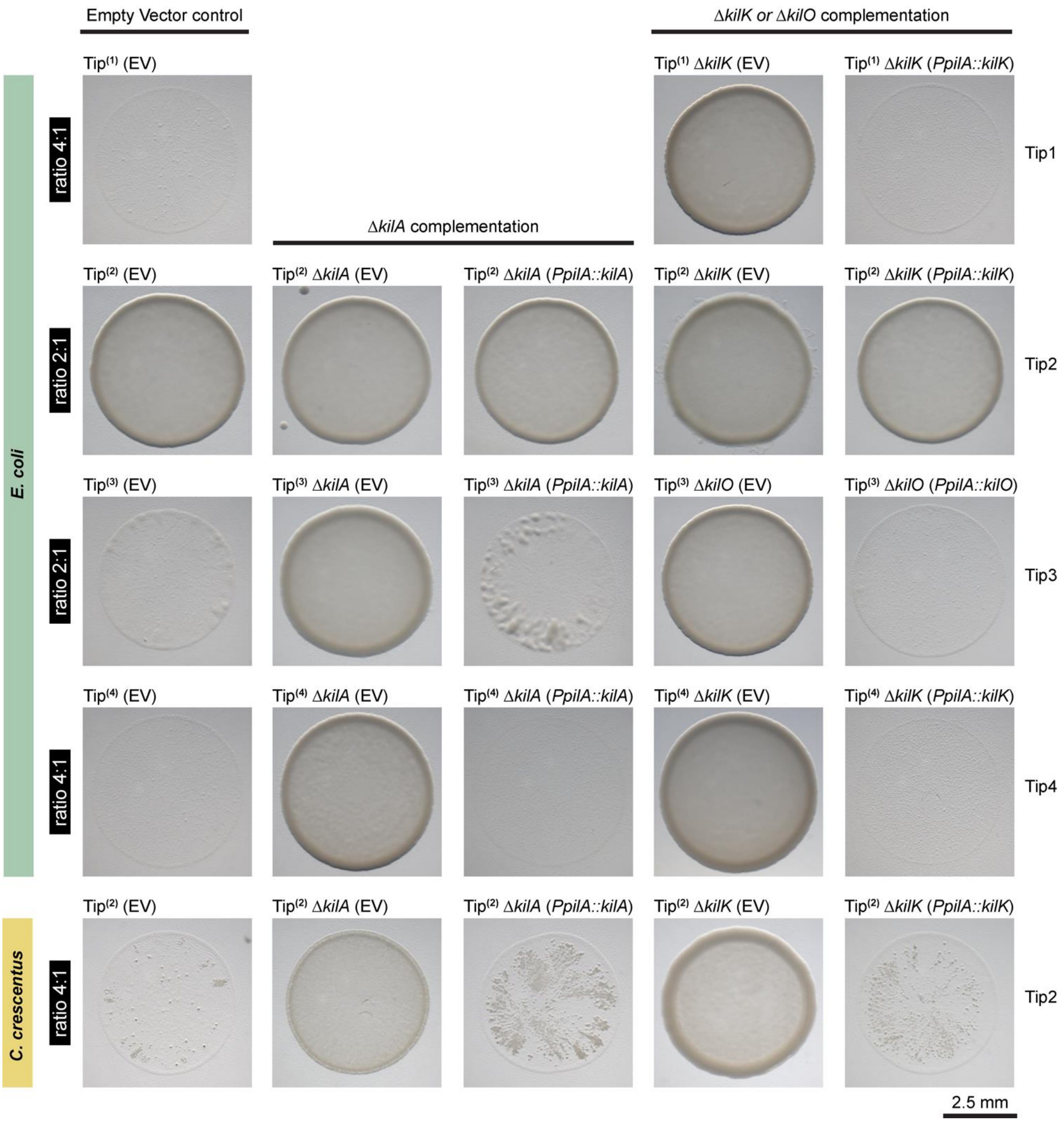

Supplementary Figure 16. The different Δ*kilA*, Δ*kilK* and Δ*kilO* strains can be ectopically complemented for predation.
Representative images of prey colonies after 24 hours of predation by the different complemented strains. Wild-type alleles of *kilA, kilK* or *kilO* were cloned into the pSWU19 plasmid and constitutively expressed under the control of the *pilA* promoter (*PpilA*). Strains carrying the empty pSWU19-*PpilA* vector (EV) were used as controls. We noticed that the pSWU19 plasmid slightly reduces the predation efficiency of the single-Tip expressing strains. To address this issue, the prey-to-predator ratios were readjusted to restore a full predation efficiency in these strains. The specific ratios used to test each single-Tip expressing strains are indicated on the left. The predation assays were performed on CF 1.5% agar plates supplemented with 0.075% glucose using *E. coli* as prey. Since the complemented Tip2-expressing strains still presented a strong predation defect under these conditions, a prey cell with a higher susceptibility to Tip2 predation was used. Predation on *C. crescentus* confirmed the successful complementation of the different Tip2-expressing strains. To make this figure, representative images of the prey colonies were taken after 24 hours of predation. All the *Myxococcus* strains used in this assay were motile.

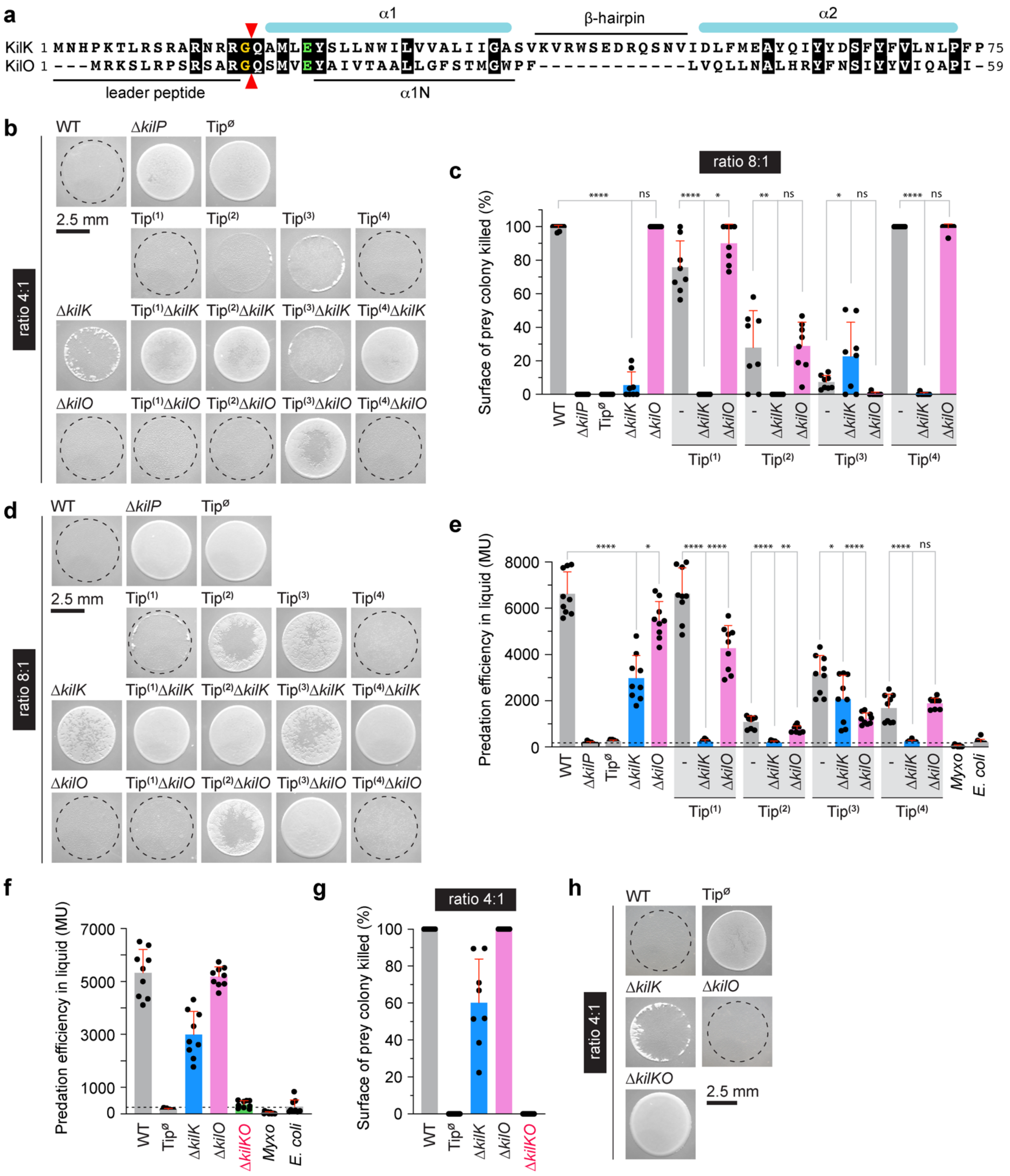

Supplementary Figure 17. The Flp minor pilins KilK and KilO are functionally associated with the Tip complexes.
a Amino acid sequence alignment of KilK (Mxan_4655) and KilO (Mxan_1150). The predicted cleavage site of the leader peptide regions is indicated by two red triangles. The hydrophobic α1N region is also delimited. Predicted secondary structures are reported above the alignment. **b** This panel shows representative pictures used to measure the surface of *E. coli* colony killed by the different Δ*kilK and* Δ*kilO* mutants after 24 hours. In this assay, prey and predator cells were mixed to a 4:1 ratio and spotted on CF 1.5% agar plates supplemented with 0.075% glucose. All the *Myxococcus* strains used in this assay were motile. **c** The predation efficiency of the different Δ*kilK and* Δ*kilO* mutants was also quantified on solid surfaces at an 8:1 prey-to-predator ratio. **d** This panel shows representative pictures used to quantify the predation efficiencies reported in panel **c**. **e** The predation efficiency of the different Δ*kilK and* Δ*kilO* mutants was also evaluated in liquid CF after 24 hours of incubation. This graph represents the kinetics of CPRG hydrolysis catalyzed by the β-Galactosidase released in the supernatant from lysed *E. coli*. *Myxococcus* and *E. coli* alone were used as negative controls. **f** The predation efficiency of a Δ*kilKO* double mutant was evaluated in liquid CF after 24 hours of incubation. **g** The Δ*kilKO* predation efficiency was also quantified on solid surfaces at an 8:1 prey-to-predator ratio. **h** This panel shows representative pictures used to quantify the predation efficiencies reported in panel **g**. In all graphs, error bars represent the standard deviation of the mean. In panels **c** and **g**, these experiments were performed independently four times, each data point representing a prey colony (*n*= 8 per strain). In panels **e** and **f**, three independent experiments were performed in triplicate (*n*= 9 per strain). In panels **c** and **e**, statistics were determined using ordinary one-way ANOVA followed by Dunnett's multiple comparisons test. Significance: $^{ns}P>0.05$, $^{*}P<0.05$, $^{**}P<0.01$, $^{****}P<0.0001$. Source data and exact *p*-values are provided as a Source Data file.

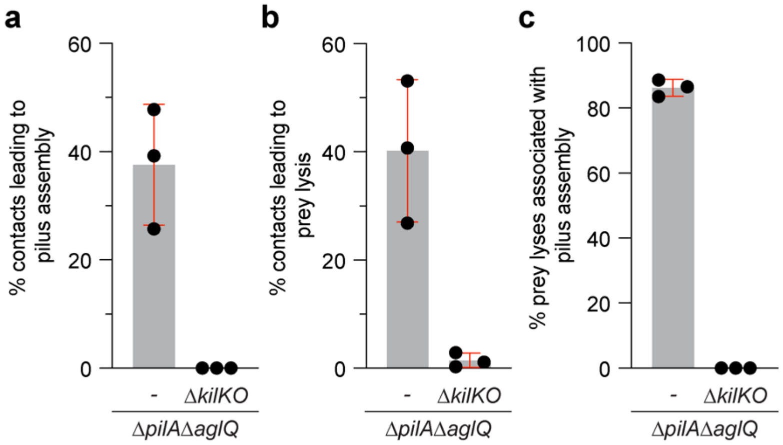

Supplementary Figure 18. The KilK and KilO minor pilins are required for KilP polymerization.
a The percentage of contacts with *E. coli* leading to KilP^{A96C} polymerization was evaluated in non-motile strains carrying or not a *kilKO* double deletion. **b** In these strains, the percentage of contacts leading to *E. coli* lysis was also estimated. **c** The percentage of *E. coli* lyses associated with KilP^{A96C} polymerization was determined as well. In panels **a** and **b**, the number of contacts with *E. coli* used for quantification was *n*= 1025 for Δ*pilA*Δ*aglQ* and *n*= 1101 for Δ*pilA*Δ*aglQ*Δ*kilKO*. In panel **c**, the number of prey lysis events used for quantification was *n*= 410 for Δ*pilA*Δ*aglQ* and *n*= 17 for Δ*pilA*Δ*aglQ*Δ*kilKO*. These quantifications were obtained from three independent 30-minute microscopy movies. Error bars represent the standard deviation of the mean. Source data are provided as a Source Data file.

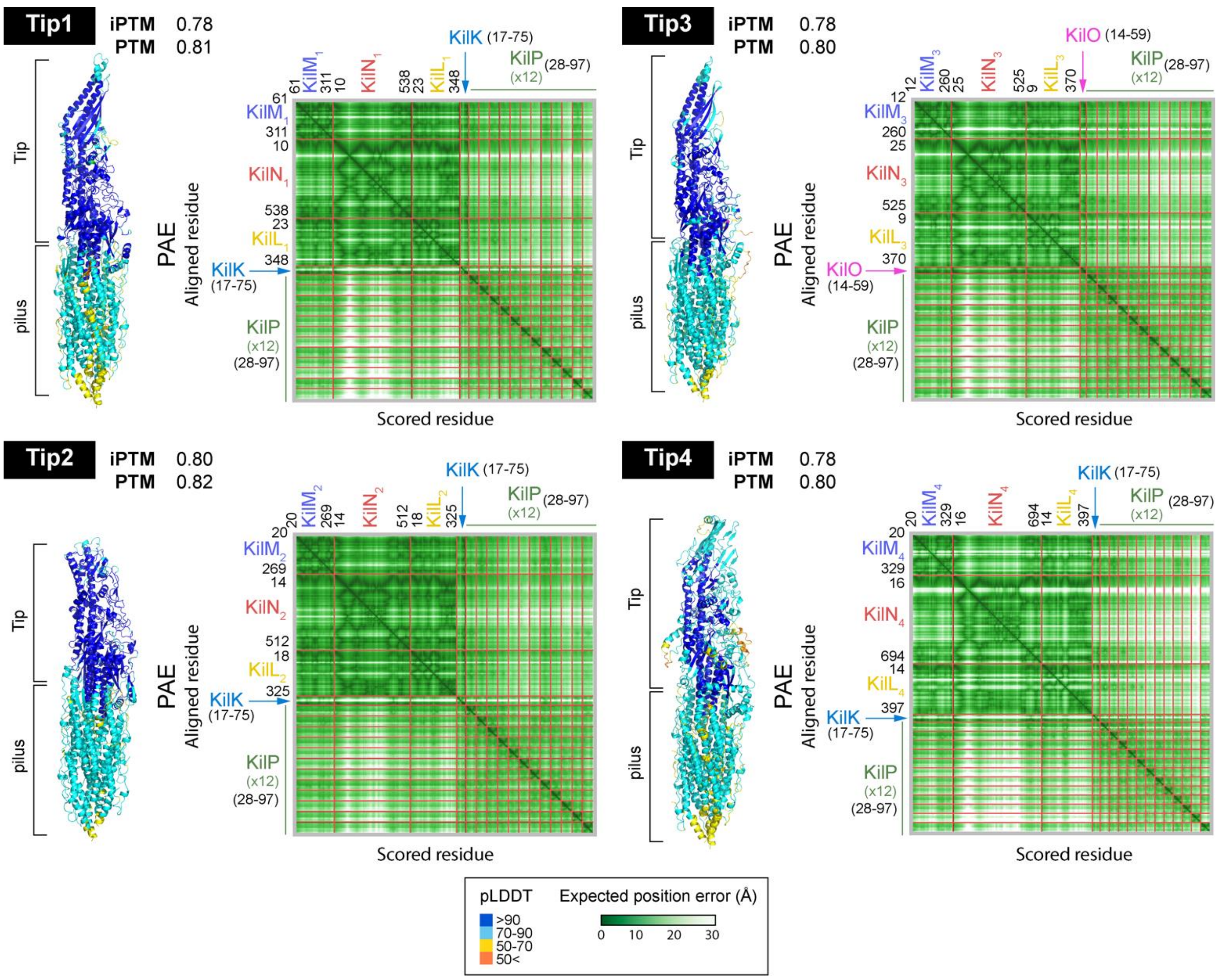

Supplementary Figure 19. pTM scores, ipTM scores, pLDDT and PAE of the different Tip models in complex with KilP, KilK or KilO.
To generate these AlphaFold predictions, the leader peptide regions were systematically removed from the major and minor pilin amino acid sequences. The predicted structures are color-coded based on the pLDDT corresponding to a per-residue model confidence score: disordered (50<); low confidence (50-70); good confidence (70-90); high confidence (>90). PAE scores: low PAE for residue pairs x, y from two different proteins or domains indicates that AlphaFold predicted well-defined relative positions and orientations for these proteins or domains. On the contrary, if PAE is high, then the relative positions and/or orientations of these proteins or domains in the 3D structure are uncertain and should not be interpreted. A pTM score above 0.5 means the overall predicted fold for the complex might be similar to the true structure. ipTM measures the accuracy of the predicted relative positions of the subunits within the complex. Values higher than 0.8 represent confident high-quality predictions.

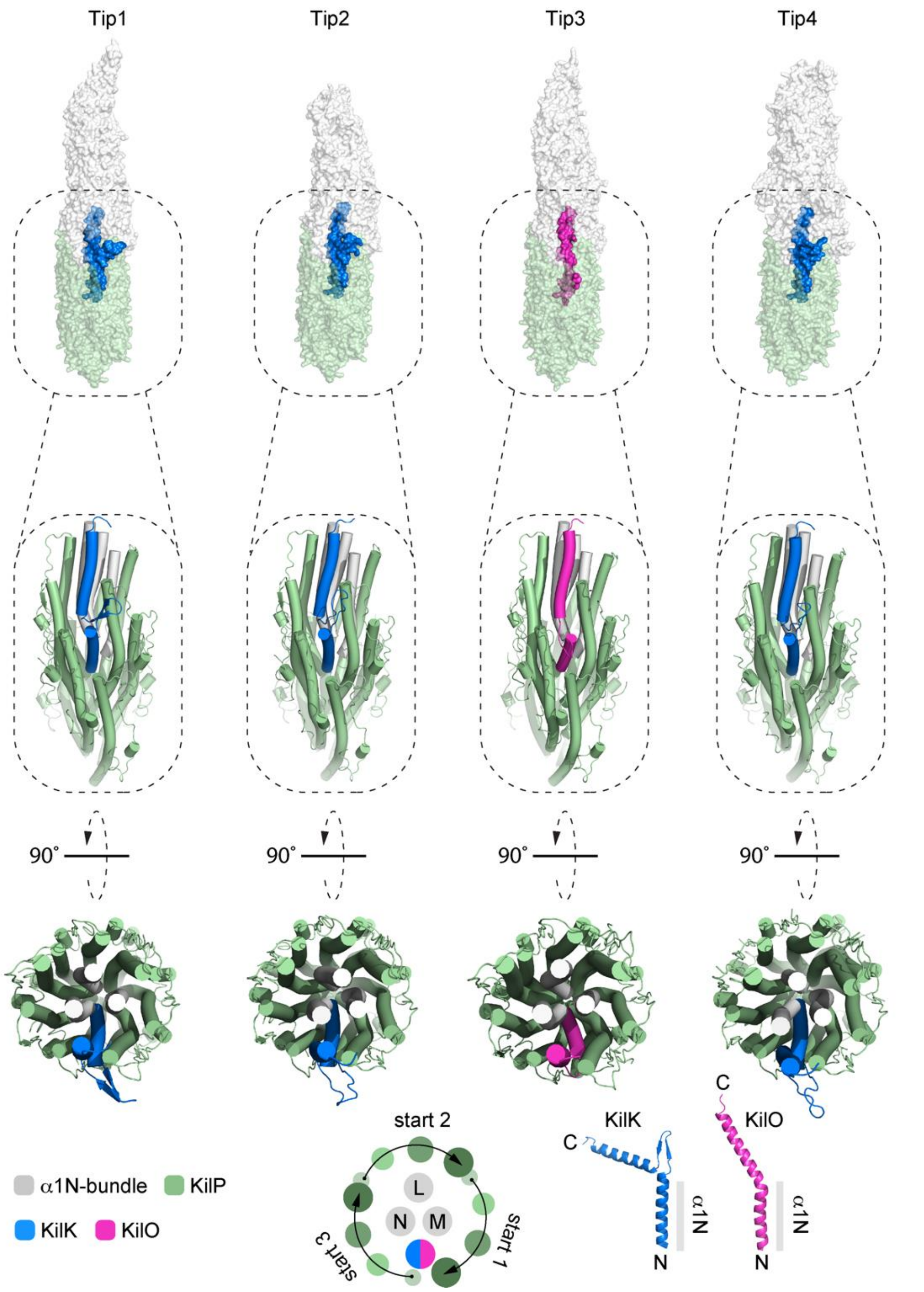

Supplementary Figure 20. KilK and KilO are structural components of the Kil pilus.
In all AlphaFold predictions, KilK and KilO are positioned at the interface between the Tip (in light grey) and the extremity of the KilP pilus (in green). The N-terminal region of these two minor pilins is deeply buried within the KilP pilus, while the C-terminal region is inserted in a hydrophobic pocket constituted by KilM, KilN and KilP. The "zoomed-in" views correspond to the region of interaction between a Tip and the pilus. For clarity, it only shows the α1N-bundle (in grey) surrounded by multiple copies of KilP (in green) and one copy of KilK or KilO (in blue and pink, respectively). A cartoon representation of the different α-helices constituting the junction between a Tip and the pilus extremity is shown at the bottom of the panel. AlphaFold predictions of KilO and KilK monomers are also provided. To generate these AlphaFold predictions, the leader peptide regions were systematically removed from the major and minor pilin amino acid sequences.

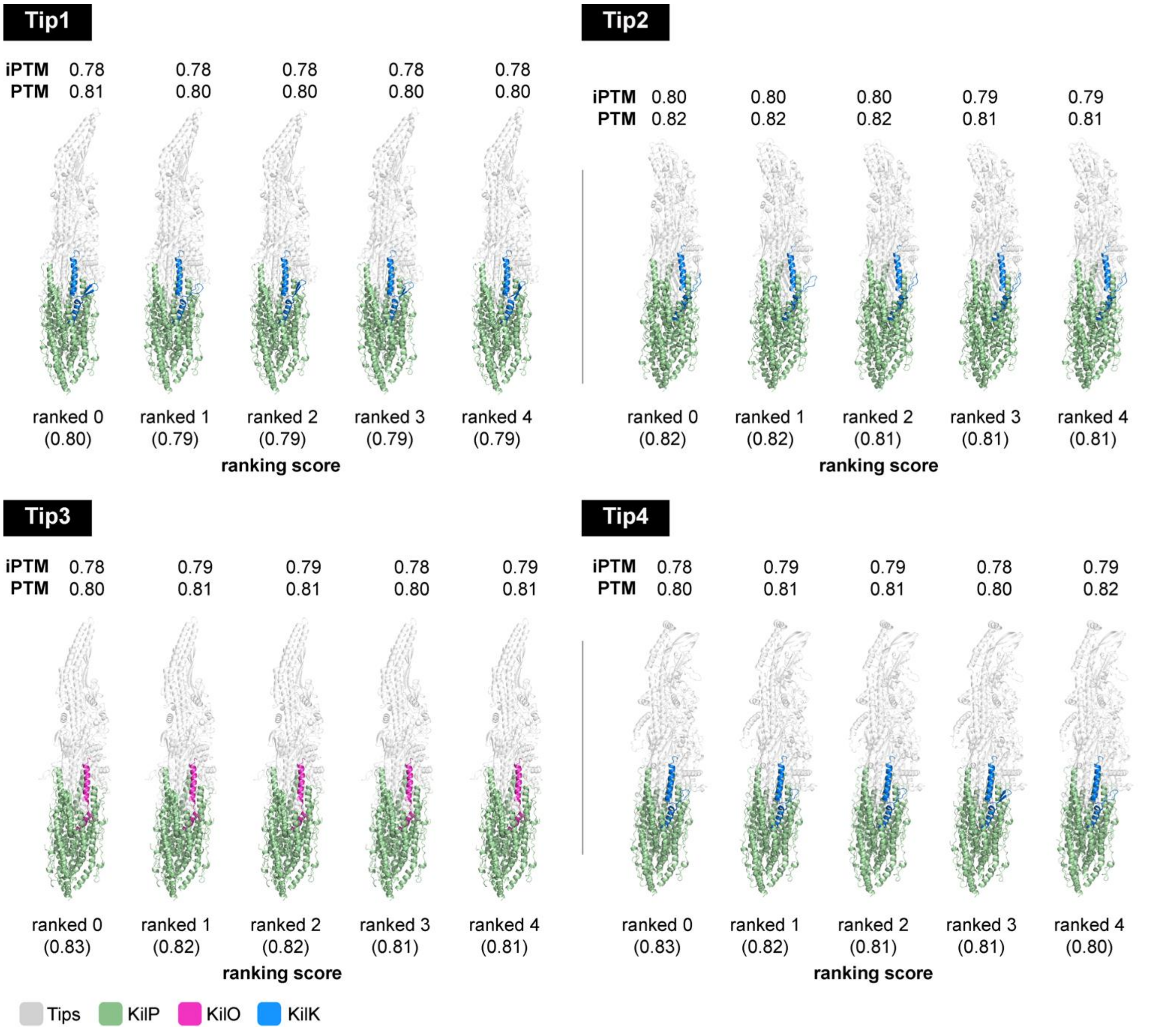

Supplementary Figure 21. Ranked AlphaFold predictions of the Tips in complex with KilP (x12) and KilK or KilO.
Within the different complexes (ranked by prediction accuracy), KilK or KilO consistently occupy the same position. The corresponding ipTM and pTM scores are indicated above each complex. The ranking score is reported below. To generate these predictions, the leader peptide regions were systematically removed from the major and minor pilin amino acid sequences.

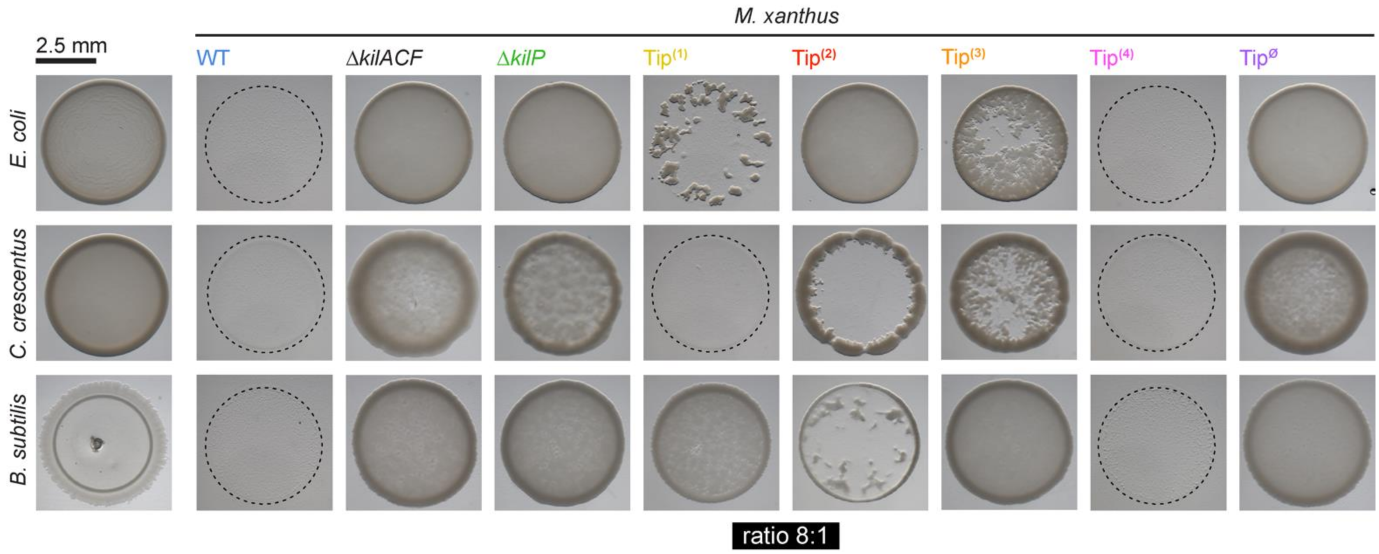

Supplementary Figure 22. Predation against diverse prey species has varying Tip requirements.
The efficiency of the single-Tip expressing strains to kill diverse bacterial species was evaluated by quantifying the number of prey-CFUs that survived to predation after 0, 8 and 24 hours of incubation. In this assay, prey and predator cells were mixed to an 8:1 ratio and spotted on CF 1.5% agar plates supplemented with 0.075% glucose. Representative pictures of the prey colonies were taken after 24 hours of predation. All the *Myxococcus* strains used in this assay were motile.

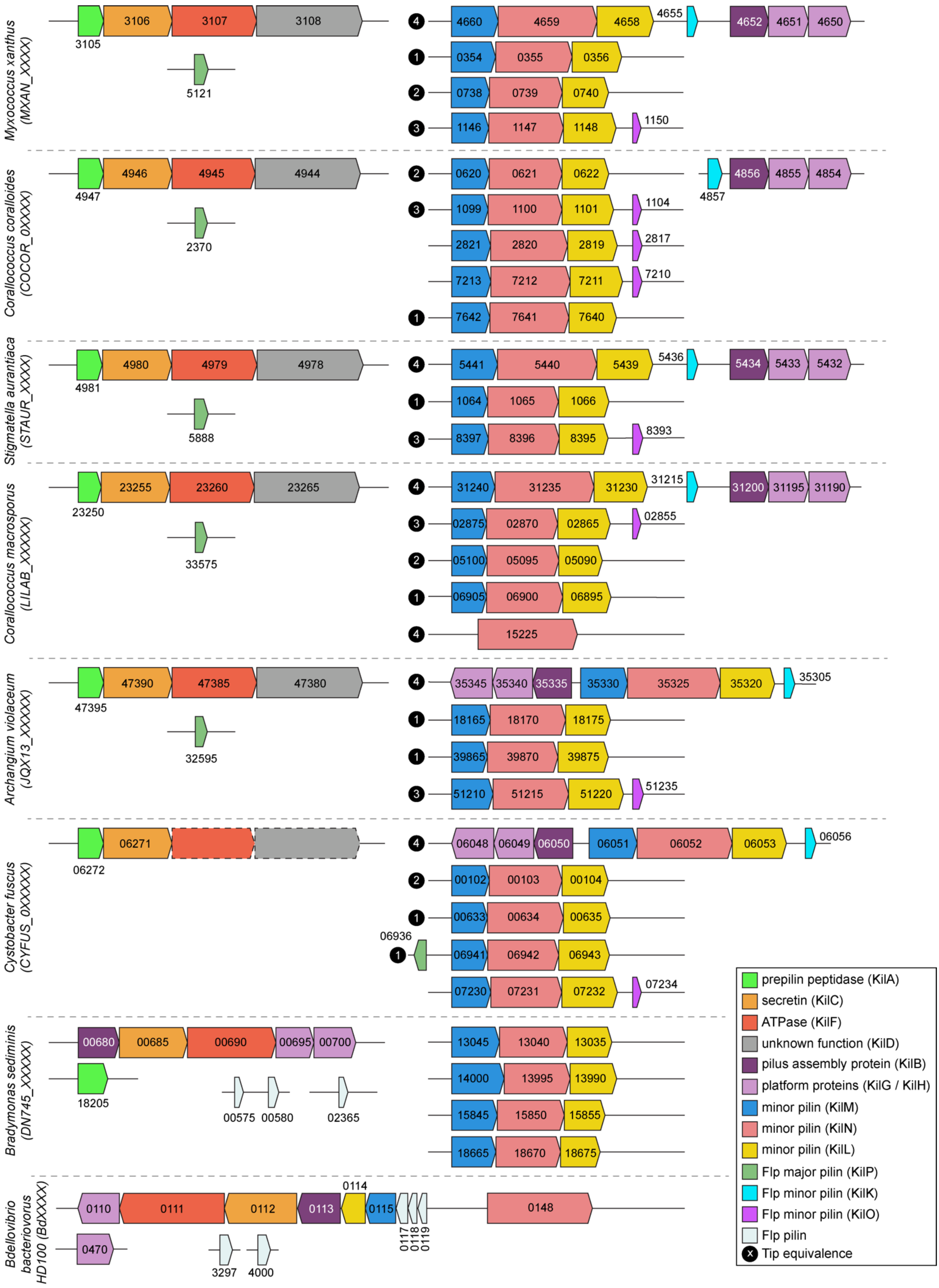

Supplementary Figure 23. The synteny of *kil*- and *tip*-orthologous genes is explored across different predatory bacteria. All the genes are color-coded according to their KEGG annotation and the *kil*-gene equivalence is also provided. In *C. fuscus*, genes encoding KilF and KilD are present but not annotated. When possible, the Tip equivalence is provided for each *kilLMN*-like gene cluster. See Supplementary Fig. 25 for Alphafold predictions corresponding to the different Tip-like complexes.

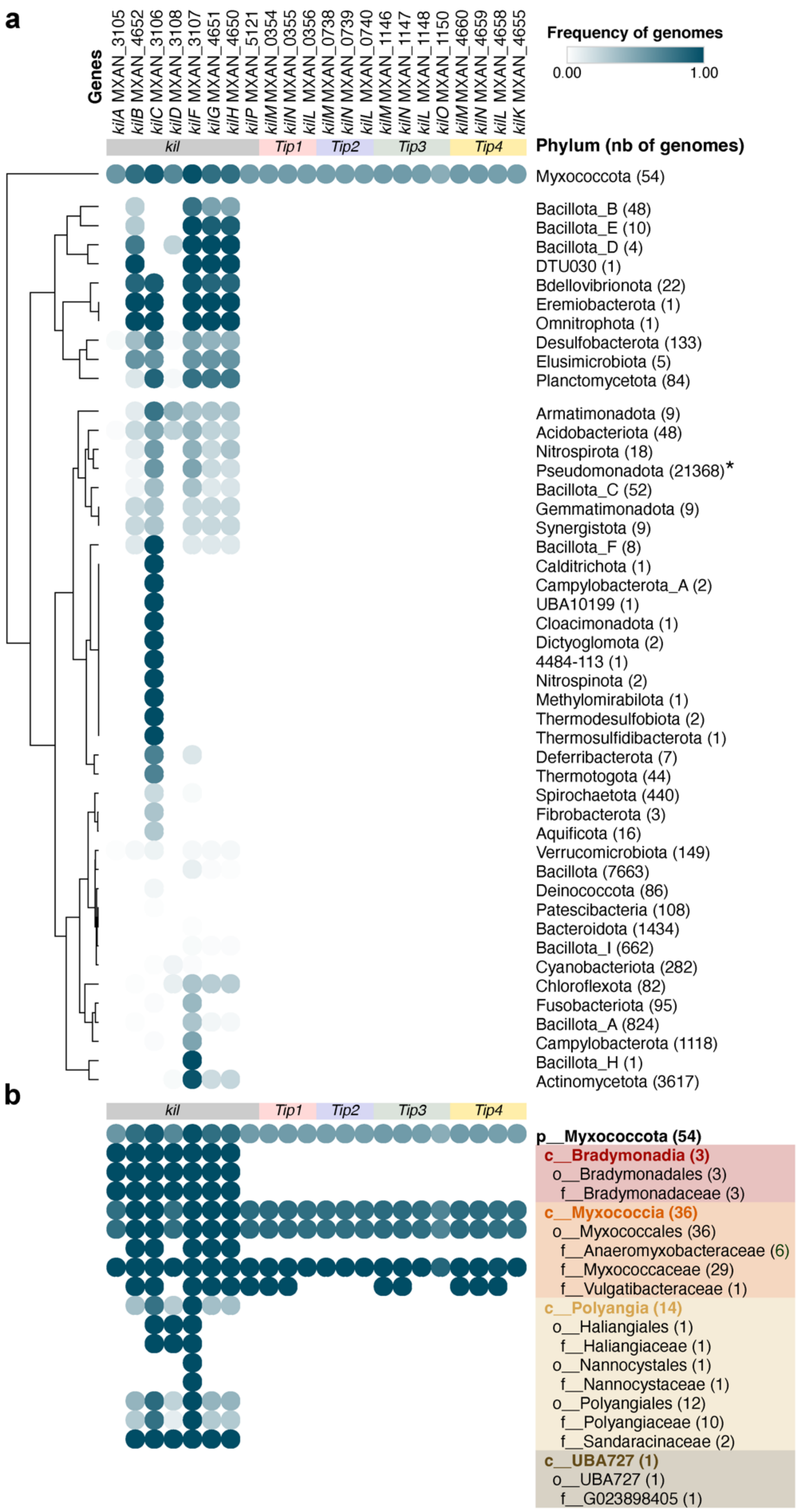

Supplementary Figure 24. Tad pili are genetically associated with Tip homologs in other bacterial species.
a Heatmap representing the frequency of complete genomes per phylum in which at least one *kil*- or *tip*-homologous gene sequence was detected using the HMM profiles built in this study. **b** Heatmap illustrating the frequency of complete genomes per family of the *Myxococcota* phylum containing at least one *kil*- or *tip*-homologous gene sequence, identified using the same HMM profiles. The classification follows the GTDB r207 taxonomy (https://gtdb.ecogenomic.org). Data were organized using hierarchical clustering analysis (Euclidean distance clustering algorithm) based on their pairwise distances. Details are given in Supplementary Data 1. *Note: homologs of certain *tip* genes were detected in 14 *Pseudomonadota* genomes, but are not visible in the figure as they represent a low frequency. Homologs were detected in 47 phyla out of 64 represented by the 38,791 genomes. P= phylum; C= class; O= order; F= family. Supplementary Data 1 provides a list of complete genomes (NCBI RefSeq database, April 2024) in which at least one *kil*- / *tip*-homologous gene sequence was detected using the HMM profiles built from the multiple sequences alignments of orthologues identified with blast and Foldseek searches in this study.

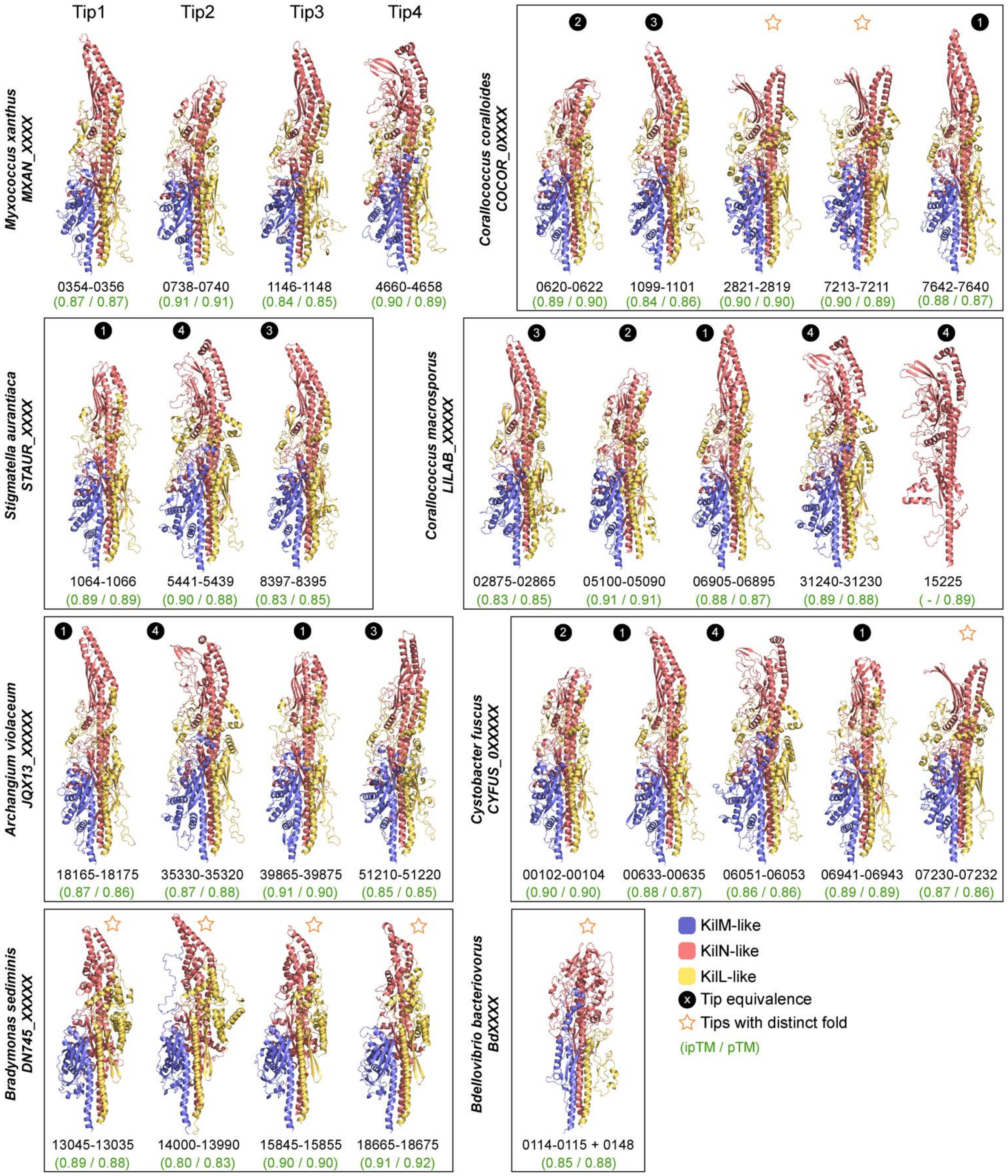

Supplementary Figure 25. Alphafold predictions of the Tip-like complexes found in diverse predatory bacteria.
To generate these predictions, the leader peptide regions were systematically removed from the corresponding minor pilin amino acid sequences. The gene numbers (in black) of the proteins constituting the Tips are provided below the predictions. When possible, the Tip equivalence is indicated for each KilLMN-like complex. The corresponding ipTM and pTM scores (in green) are reported below each complex.

Bacterial species	Gene number	Annotation	Function
Bordetella pertussis Tohama I (human pathogen)	*BP1991*	Flp / PilA	Flp pilin
	BP1992	CpaA / TadV	Prepilin peptidase
	BP1993	CpaJK / TadEF	Minor pilin
	BP1994	CpaJK / TadEF	Minor pilin
	BP1995	CpaB / RcpC	Inner membrane subunit
	BP1996	CpaC / RcpA	Secretin
	BP1997	CpaE / TadZ	ATPase-receiver protein
	BP1998	CpaF / TadA	Motor ATPase
	BP1999	CpaG / TadB	Platform protein
	BP2000	CpaH / TadC	Platform protein
	BP2001	CpaO / TadD	Pilotin
	BP2003	CpaL / TadG	Large minor pilin
	BP2525	CpaG / TadB	Platform protein
	BP2525A	CpaF / TadA	Motor ATPase
	BP2526	CpaC / RcpA	Secretin
	BP2527	CpaB / RcpC	Inner membrane subunit
	BP2528A	CpaJK / TadEF	Minor pilin
	BP2529	CpaL / TadG	Large minor pilin
	BP2530	Flp / PilA	Flp pilin
Pseudomonas aeruginosa PAO1 (opportunistic and nosocomial pathogen in humans)	*PA4294*	CpaJK / TadEF	Minor pilin
	PA4295	CpaA / FppA	Prepilin peptidase
	PA4297	CpaL / TadG	Large minor pilin
	PA4299	CpaO / TadD	Pilotin
	PA4300	CpaH / TadC	Platform protein
	PA4301	CpaG / TadB	Platform protein
	PA4302	CpaF / TadA	Motor ATPase
	PA4303	CpaE / TadZ	ATPase-receiver protein
	PA4304	CpaC / RcpA	Secretin
	PA4305	CpaB / RcpC	Inner membrane subunit
	PA4306	Flp / PilA	Flp pilin
Burkholderia gladioli BSR3 (plant pathogen and opportunistic pathogen in humans)	*bgla_1g18150*	Flp / PilA	Flp pilin
	bgla_1g18160	CpaA / TadV	Prepilin peptidase
	bgla_1g18170	CpaJK / TadEF	Minor pilin
	bgla_1g18180	CpaB / RcpC	Inner membrane subunit
	bgla_1g18190	CpaC / RcpA	Secretin
	bgla_1g18200	CpaE / TadZ	ATPase-receiver protein
	bgla_1g18210	CpaF / TadA	Motor ATPase
	bgla_1g18220	CpaG / TadB	Platform protein
	bgla_1g18230	CpaH / TadC	Platform protein
	bgla_1g18240	CpaO / TadD	Pilotin
	bgla_1g18260	CpaL / TadG	Large minor pilin
	bgla_2g00380	CpaA / TadV	Prepilin peptidase
	bgla_2g00390	Flp / PilA	Flp pilin
	bgla_2g00400	CpaL / TadG	Large minor pilin
	bgla_2g00410	CpaJK / TadEF	Minor pilin
	bgla_2g00430	Flp / PilA	Flp pilin
	bgla_2g00440	CpaB / RcpC	Inner membrane subunit
	bgla_2g00450	CpaC / RcpA	Secretin
	bgla_2g00460	CpaF / TadA	Motor ATPase
	bgla_2g00470	CpaG / TadB	Platform protein
	bgla_2g00480	CpaH / TadC	Platform protein
	bgla_2g00500	CpaJK / TadEF	Minor pilin
Stenotrophomonas maltophilia JV3 (opportunistic and nosocomial pathogen in humans)	*BurJV3_0862*	CpaJK / TadEF	Minor pilin
	BurJV3_0864	CpaH / TadC	Platform protein
	BurJV3_0865	CpaG / TadB	Platform protein
	BurJV3_0866	CpaF / TadA	Motor ATPase
	BurJV3_0867	CpaC / RcpA	Secretin
	BurJV3_0868	CpaB / RcpC	Inner membrane subunit
	BurJV3_0869	Flp / PilA	Flp pilin
	BurJV3_0871	CpaJK / TadEF	Minor pilin
	BurJV3_0873	CpaL / TadG	Large minor pilin
	BurJV3_0874	Flp / PilA	Flp pilin

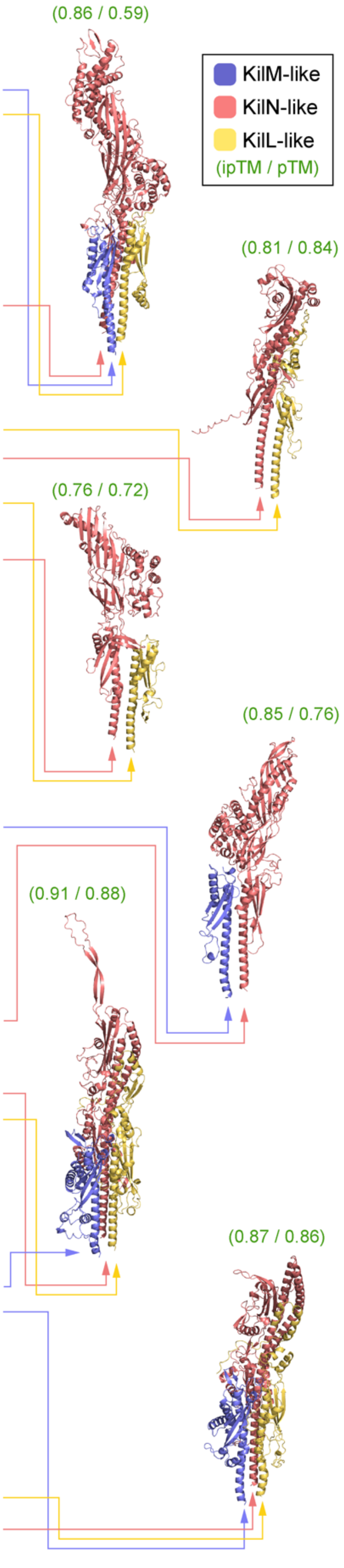

Supplementary Figure 26. Examples of Tip-like pilins present in *tad* loci of distantly related bacterial species.
This table compiles the *tad*-gene clusters found in different bacterial species, along with their KEGG annotations and predicted functions. The structures of the TadEFG complexes were predicted with AlphaFold. The corresponding ipTM and pTM scores (in green) are indicated above each prediction. To generate these models, the leader peptide regions were systematically removed from the amino acid sequences of the corresponding minor pilins.
