# Supplementary material for: Tad pili with adaptable tips mediate contact-dependent killing during bacterial predation
Source: Nat Commun. 2025 May 13;16:4425. doi: 10.1038/s41467-025-58967-0 (PMC12075869; doi:10.1038/s41467-025-58967-0)
Supplement: Supplementary file 8 — Reporting Summary [file 41467_2025_58967_MOESM8_ESM.pdf]

nature portfolio

Corresponding author(s): Julien Herrou
Tâm Mignot

Last updated by author(s): Feb 10, 2025

Reporting Summary

Nature Portfolio wishes to improve the reproducibility of the work that we publish. This form provides structure for consistency and transparency in reporting. For further information on Nature Portfolio policies, see our Editorial Policies and the Editorial Policy Checklist.

Statistics

For all statistical analyses, confirm that the following items are present in the figure legend, table legend, main text, or Methods section.

n/a	Confirmed	
☐	☒	The exact sample size (*n*) for each experimental group/condition, given as a discrete number and unit of measurement
☐	☒	A statement on whether measurements were taken from distinct samples or whether the same sample was measured repeatedly
☐	☒	The statistical test(s) used AND whether they are one- or two-sided<br>*Only common tests should be described solely by name; describe more complex techniques in the Methods section.*
☒	☐	A description of all covariates tested
☒	☐	A description of any assumptions or corrections, such as tests of normality and adjustment for multiple comparisons
☐	☒	A full description of the statistical parameters including central tendency (e.g. means) or other basic estimates (e.g. regression coefficient) AND variation (e.g. standard deviation) or associated estimates of uncertainty (e.g. confidence intervals)
☐	☒	For null hypothesis testing, the test statistic (e.g. *F*, *t*, *r*) with confidence intervals, effect sizes, degrees of freedom and *P* value noted<br>*Give P values as exact values whenever suitable.*
☒	☐	For Bayesian analysis, information on the choice of priors and Markov chain Monte Carlo settings
☒	☐	For hierarchical and complex designs, identification of the appropriate level for tests and full reporting of outcomes
☒	☐	Estimates of effect sizes (e.g. Cohen's *d*, Pearson's *r*), indicating how they were calculated

Our web collection on statistics for biologists contains articles on many of the points above.

Software and code

Policy information about availability of computer code

Data collection

The following softwares and servers were used for data collection:
- Bacterial microscopy was performed with NIS Elements AR 52.42.06 software (Nikon).
- AlphaFold structures were generated using ColabFold pipeline and AlphaFold3 server.
- Enzymatic activities were recorded with a Tecan plate reader using SparkControl V3.1 SP1 acquisition software.
- The list of 38,791 latest complete genomes assemblies referenced in the GTDB taxonomy was used with the NCBI Datasets command-line tools (CLI) ncbi-datasets-cli-16.19.0 to download from the NCBI RefSeq database all coding sequences in the corresponding assemblies.

Data analysis

The following softwares and servers were used for data analysis:
- Image analyses were performed with ImageJ2-Fiji 2.14.0 / 1.54f (https://imagej.net/software/fiji/)
- Protein structure analyses were performed with PyMOL 3.0.3 (https://www.pymol.org/)
- Protein structure analyses were performed with ChimeraX 1.7rc202311180239 (https://www.cgl.ucsf.edu/chimerax/)
- Graphs and statistical analyses were performed using GraphPad Prism 10.3.1 (https://www.graphpad.com/)
- Protein structure comparisons were performed with DALI server (http://ekhidna2.biocenter.helsinki.fi/dali/)
- Amino acid sequence alignments were performed using Uniprot server (https://www.uniprot.org/align)
- Protein contact maps were generated using Mapiya server (https://mapiya.lcbio.pl/)
- Structural homologs were found using FoldSeek search server (https://search.foldseek.com/search)
- Gene orthologs and paralogs were found using the KEGG server (https://www.kegg.jp/)
- Hidden Markov model (HMM) profiles were built with HMMER v3.489 (http://hmmer.org)
- Multiple sequence alignments (MSA) were generated with MAFFT v7.490 (L-INS-i method)

- Gene coding sequences were translated using a Python script with Biopython v 1.78 (https://github.com/biopython/), and PyHMMER 0.8.292 was used to extract the best hits with a minimal E-value of 10^25

For manuscripts utilizing custom algorithms or software that are central to the research but not yet described in published literature, software must be made available to editors and reviewers. We strongly encourage code deposition in a community repository (e.g. GitHub). See the Nature Portfolio guidelines for submitting code & software for further information.

Data

Policy information about availability of data

All manuscripts must include a data availability statement. This statement should provide the following information, where applicable:

- Accession codes, unique identifiers, or web links for publicly available datasets
- A description of any restrictions on data availability
- For clinical datasets or third party data, please ensure that the statement adheres to our policy

The authors declare that all data supporting this study are available within the article, its Supplementary Information file or in the Source Data files. Source data files are provided with this paper.

Research involving human participants, their data, or biological material

Policy information about studies with human participants or human data. See also policy information about sex, gender (identity/presentation), and sexual orientation and race, ethnicity and racism.

Reporting on sex and gender	Not applicable
Reporting on race, ethnicity, or other socially relevant groupings	Not applicable
Population characteristics	Not applicable
Recruitment	Not applicable
Ethics oversight	Not applicable

Note that full information on the approval of the study protocol must also be provided in the manuscript.

Field-specific reporting

Please select the one below that is the best fit for your research. If you are not sure, read the appropriate sections before making your selection.

☒ Life sciences ☐ Behavioural & social sciences ☐ Ecological, evolutionary & environmental sciences

For a reference copy of the document with all sections, see nature.com/documents/nr-reporting-summary-flat.pdf

Life sciences study design

All studies must disclose on these points even when the disclosure is negative.

Sample size	No statistical methods were used to predetermine the sample size. The sample size was determined based on our expertise in bacterial cell biology. Generally, at least three biological replicates were used, as it is standard practice.<br>Single cell image analysis was performed with a high number of cells from at least three biological replicates.
Data exclusions	No data were excluded.
Replication	All experiments were successfully replicated. The nature and number of replicates is indicated in the corresponding figure legend.<br>All image analyses were performed on a representative set of images.
Randomization	The experiments were not randomized, since there was no allocation into subgroups.
Blinding	No blinding was performed because the acquisition and analysis required human intervention

Reporting for specific materials, systems and methods

We require information from authors about some types of materials, experimental systems and methods used in many studies. Here, indicate whether each material, system or method listed is relevant to your study. If you are not sure if a list item applies to your research, read the appropriate section before selecting a response.

Materials & experimental systems

n/a	Involved in the study
☒	☐ Antibodies
☒	☐ Eukaryotic cell lines
☒	☐ Palaeontology and archaeology
☒	☐ Animals and other organisms
☒	☐ Clinical data
☒	☐ Dual use research of concern
☒	☐ Plants

Methods

n/a	Involved in the study
☒	☐ ChIP-seq
☒	☐ Flow cytometry
☒	☐ MRI-based neuroimaging

Plants

Seed stocks	Not applicable
Novel plant genotypes	Not applicable
Authentication	Not applicable
